# TSC-associated microglial hyperactivity: enhanced calcium signaling, metabolism, and phagocytosis

**DOI:** 10.1007/s00401-026-02986-8

**Published:** 2026-02-13

**Authors:** Rozemarijn S. Kalf, Mark J. Luinenburg, Giulia Dematteis, Mirte Scheper, Jasper J. Anink, Giulia Cavallo, Andrea Mattarei, Wim Van Hecke, Angelika Mühlebner, Laura Tapella, James D. Mills, Dmitry Lim, Eleonora Aronica

**Affiliations:** 1 Department of (Neuro) Pathology, Amsterdam Neuroscience, Amsterdam UMC location University of Amsterdam, Meibergdreef 9, Amsterdam, The Netherlands; 2 Department of Pharmaceutical Sciences, Università del Piemonte Orientale “Amedeo Avogadro”, Novara, Italy; 3 Department of Pharmaceutical and Pharmacological Sciences, University of Padua, Padua, Italy; 4 Department of Pathology, University Medical Center Utrecht, Utrecht, The Netherlands; 5 Laboratory for Molecular Biodiscovery, Department of Pharmaceutical and Pharmacological Sciences, KU Leuven, Leuven, Belgium

**Keywords:** Tuberous sclerosis complex, Epilepsy, Calcium signalling, Phagocytosis, Microglia

## Abstract

Tuberous sclerosis complex (TSC**)** is a multisystem genetic disorder with prominent neurological manifestations, most notably epilepsy, and is frequently accompanied by a wide range of neuropsychiatric comorbidities. Hyperactivation of the mechanistic target of rapamycin (mTOR) pathway plays a central role in TSC pathology, disrupting both general brain development and specific molecular processes such as metabolism. While much attention has focused on neurons and astrocytes in these TSC-related alterations, the contribution of microglia remains relatively underexplored. In this study, we first analysed the transcriptomic profiles from resected TSC brain tissue and identified evidence of calcium (Ca^2+^) dysregulation in TSC microglia. In order to investigate the functional consequences, we then examined induced pluripotent stem cell (iPSC) derived microglia-like (iMGL) cells from TSC patients. Our findings reveal that these iMGL cells displayed markedly altered Ca^2^⁺ signalling, characterized by impaired store-operated calcium entry (SOCE) and an increase in mitochondrial Ca^2^⁺ uptake. These changes are accompanied by elevated mitochondrial respiratory activity, suggesting a shift in metabolic state. In addition, TSC iMGL cells displayed increased phagocytic activity and an altered inflammatory responsiveness, consistent with a dysregulated microglial activation state. Supporting these functional alterations in iMGL cells, transcriptomic analysis of TSC brain tissue revealed upregulation of several genes associated with lipid metabolism, phagocytosis, and innate immune activation, with partial overlap with stage 2 disease-associated microglia (DAM)-like programs. Together these findings suggest that microglial dysfunction may represent a relevant component of TSC pathophysiology.

**Supplementary Information:**

The online version contains supplementary material available at 10.1007/s00401-026-02986-8.

## Introduction

Tuberous sclerosis complex (TSC) is a rare genetic disorder characterized by the formation of benign tumors across multiple organs, including the eyes, kidneys, lungs, and brain, as well as cortical lesions known as tubers [14, 69]. Tubers are developmental lesions defined by delamination within the cortical regions, along with disruptions at the grey–white matter border, and consist of dysmorphic cells, disorganized or decreased numbers of neurons, and reactive microglia and astrocytes [2, 14, 109]. In TSC, epilepsy is the most common neurological manifestation, occurring in 80–90% of affected individuals. Beyond epilepsy, TSC is frequently associated with a wide range of neuropsychiatric manifestations on behavioral, intellectual, neuropsychological, academic, psychiatric and psychosocial levels, collectively referred to as TSC-associated neuropsychiatric disorders (TANDs) [13, 15, 98]. TSC arises from the loss-of-function mutations in the tumor suppressor genes *TSC1* or *TSC2*, encoding the proteins hamartin and tuberin, respectively. These proteins form a complex that negatively regulates the mechanistic target of rapamycin complex 1 (mTORC1), a critical kinase involved in cellular growth and metabolism [14, 58]. Mutations in *TSC1* or *TSC2* result in hyperactivation of the mTOR signaling pathway [12, 14]. Recent neuropathological, molecular, and cellular studies have highlighted hallmark features of TSC pathology, including disruptions in neurotransmission, synaptic plasticity, energy metabolism, neuronal support mechanisms, and early neuroinflammation [2, 27, 89, 109]. While astrocytes have received considerable attention [56, 82], the role of microglia in TSC remains understudied.

Microglia, the primary immune cells of the central nervous system (CNS), have a distinct developmental origin, arising from yolk sac progenitors rather than hematopoietic precursors [19, 32, 44]. They play an essential role in maintaining CNS homeostasis by continuously surveilling their microenvironment and dynamically responding to neural injury or aberrant activity [19, 95]. Microglial activation is a key factor in epilepsy and associated comorbidities, correlating in TSC with white matter pathology and cognitive deficits [1, 21, 64]. Activated microglia release inflammatory mediators that modulate ion channels and receptors, including GABAA, thereby disrupting the excitation/inhibition balance [83]. Chronic immune dysregulation is maintained by impaired resolution mechanisms and epigenetic changes, such as IL1β promotor hypomethyliation, while transcriptomic profiling reveals links to oxidative stress, mitochondrial dysfunction, and prolonged inflammatory signaling [2, 8, 28, 63].

Microglia exhibit remarkable motility, enabling precise migration to sites of immune intervention [16, 68]. Under physiological conditions, microglial calcium (Ca2⁺) signaling is minimal [10, 23, 75]. However, pathological conditions induce pronounced alterations in Ca^2^⁺ dynamics, which play key roles in microglial functions such as motility, cytokine secretion, and receptor trafficking [24, 30, 47, 52, 94, 96]. In microglia, Ca^2^⁺ signaling involves influx across the plasma membrane and release from intracellular stores, primarily the ER and mitochondria [30, 52, 53]. ER-mediated Ca^2^⁺ release is the predominant pathway, facilitated by ryanodine receptors (RyRs) and inositol 1,4,5-trisphosphate receptors (Ins(1,4,5)P_3_Rs). Store-operated Ca^2+^ entry (SOCE) replenishes ER Ca^2^⁺ stores via sarco/endoplasmic reticulum Ca^2^⁺-ATPases (SERCAs), with STIM1 and STIM2 acting as luminal ER Ca^2^⁺ sensors to activate ORAI family Ca^2^⁺ channels [62]. Additionally, mitochondria positioned near the ER, and plasma membrane Ca^2^⁺ channels efficiently take up Ca^2^⁺ via the mitochondrial calcium uniporter (MCU) in response to ER release or membrane influx, forming localized Ca^2^⁺ microdomains [11, 80, 84].

Dysregulated Ca^2^⁺ signalling has been implicated in various neurodegenerative diseases (e.g., Alzheimer’s disease (AD), Parkinson’s disease (PD)), schizophrenia, epilepsy and neurodevelopmental disorders [5, 19, 53, 61, 82, 90, 91, 101, 110]. A recent study has reported altered Ca^2^⁺ signalling in primary astrocytes isolated from TSC patients, demonstrating reduced cellular responsiveness to Ca^2^⁺ fluctuations [82]. These findings are consistent with existing evidence linking mTORC1 activation to calcium dysregulation [35, 51]. Based on this emerging evidence, we hypothesize that mTOR hyperactivation leads to Ca^2^⁺ dysregulation in microglia, a process that may contribute significantly to TSC pathophysiology and warrants further investigation. To challenge this hypothesis, we first analysed the transcriptomic profiles from resected TSC brain tissue, and identified evidence of Ca^2+^ dysregulation in TSC microglia. We then generated a cohort of seven induced pluripotent stem cell (iPSC) lines derived from patient astrocytes isolated from surgically resected lesions, alongside five control lines. A subset of these iPSCs was differentiated into iPSC-derived microglia-like (iMGL) cells, a model that has advanced the study of microglial involvement in neurodegenerative diseases such as AD and PD [25, 39, 60, 85], allowing us to assess their functional properties and investigate the role of iMGL cells in TSC pathophysiology.

In this study, we identified Ca^2+^ dysregulations in both microglia from TSC brain tissue and patient-derived iMGL cells. Moreover, TSC iMGL cells exhibited an altered inflammatory response, elevated mitochondrial respiration, and enhanced phagocytic activity. Those features indicate a hyperresponsive, metabolically active microglial state resembling the phenotype of stage 2 disease-associated microglia (DAM), as described in the context of neurodegenerative diseases [17, 43, 86, 88, 108].

## Material & methods

### Single-cell RNA sequencing (scRNA-seq)

#### Study cohort

Surgical and postmortem brain tissues were selected from the archives of the Department of Neuropathology of the Amsterdam UMC (Amsterdam, The Netherlands) and the UMC Utrecht (Utrecht, The Netherlands). Cortical brain samples were obtained from patients undergoing surgery for intractable epilepsy and diagnosed with TSC cortical tubers (*n* = 5). In addition, age- and tissue-matched autopsy control samples were collected (*n* = 3) from individuals without a history of seizures or other neurological disease. All autopsies were performed within 9 h after death. Tissue was obtained and used in accordance with the Declaration of Helsinki and the Amsterdam UMC Research Code provided by the Medical Ethics Committee and according to the Amsterdam UMC and UMC Utrecht Biobank Regulations (W21-295; 21-174). Clinical information about the brain samples is summarized in Supplementary Table 1.

#### Control and TSC scRNA-seq dataset

scRNA-seq was performed at Single Cell Discoveries (https://www.scdiscoveries.com/) according to the 10× genomics Chromium Single Cell Gene Expression Flex protocol. Prior to loading the samples, the frontal cortex tissue was cut into slices, which were fixed, and the cells were extracted. Cells were counted to ensure quality control. For each sample, 8000 cells were loaded, and the resulting sequencing libraries were prepared following a standard 10× Genomics protocol.

#### Processing and analysis of scRNA-seq data

Sample reads were aligned to the human genome GRCh38 using Cell Ranger. Filtering of empty barcodes was done in Cell Ranger. The data from all samples were loaded in R (version 4.3.1) and processed using the Seurat package (version 5.0.1). More specifically, cells with at least 1000 UMIs per cell and less than 5% mitochondrial gene content were retained for analysis. The data from all 10× libraries was merged and processed together. The merged dataset was normalized for sequencing depth per cell and log-transformed. After filtering, datasets were integrated using reciprocal principal component analysis (RPCA). The integrated data was subsequently scaled, and dimensionality reductions were performed. Clustering was computed using the FindNeighbors function (dims = 1:20) and FindClusters at a resolution of 0.5. To identify the cell types in separate clusters, FeaturePlots of marker genes for each cell type were used. To perform differential expression analysis between control and TSC samples, we performed pseudo-bulk analysis. This approach involves aggregating cells within each biological sample to create ‘pseudobulks’. Differential expression analysis was performed using the R package DESeq2 [55]. To control the false discovery rate, we applied the Benjamini–Hochberg correction, considering gene expression changes with an adjusted *p*-value < 0.05 as statistically significant. Pathway enrichment analyses were performed using the R package clusterProfiler providing a statistical analysis and visualization of GO functional profiles of genes [102, 104].

### Differentiation of iMGL cells

#### Growth factors, cytokines and preparations of reagents

All factors were stored in 5% trehalose in phosphate-buffered saline (PBS) pH 7.4, and chemical reagents were stored in dimethyl sulfoxide (DMSO). All stock solutions were stored at − 80 °C. Antibiotics are abbreviated as follows: 100 μg/mL penicillin (P), 100 μg/mL streptomycin (S).

#### Reprogramming plasmids production and purification

MIP-339-CoMiP-coLin28-2A-Nanog-IRES-eGFP and MIP-247-CoMiP-4in1-shRNAp53 were a gift from Mark Kay & Joseph Wu (Addgene plasmid #63729, #63726). Provided agar stabs were grown on 6% sucrose agar plates for 24 h at 37 °C. Clones were picked and grown in selection media (10 g/L tryptone, 5 g/L yeast extract, 6% sucrose) for 18 h at 220 RPM and 30 °C. Minipreps were performed with NucleoSpin Plasmid kits (Machery-Nagel, #740588.50) and clones were verified by restriction digestion analysis (CoMiP-247: HindIII, NotI; CoMiP-339: EcoRI, SmaI). For midipreps, cultures were inoculated 1:1000 and left to grow for 16–18 h in 80 mL production media (12.5 g/L tryptone extract, 6 g/L yeast extract, 50 g/L glycerol and 1% sucrose) at 220 RPM in 500 mL erlenmeyers. Midipreps were performed with NucleoBond Xtra Midi kits (Machery-Nagel, #740410.50). Plasmids were cleaned from endotoxin using a triton-X114 protocol [57] and purified by Zymo-Spin VI columns (Zymo Research, #C1013-10) following the manufacturer’s instruction.

#### iPSC generation

Primary derived astrocytes were isolated from surgically resected tissue from 7 TSC patients carrying heterozygous pathogenic variants in either the TSC1 or TSC2 gene (Table 1), and 5 healthy controls were derived from fetal tissue as described previously [56]. Astrocytes were maintained in astrocyte media (DMEM/F10 1:1, 10% FCS, 1 mM glutamine, P/S) at 37 °C and 5% CO_2_). For the reprogramming of astrocytes, we implemented and modified an existing protocol using MiP-339 and MIP-247 [22]. For iPSC generation, 1 × 10^7^ cells were washed with PBS and gently resuspended in 100 μL electroporation buffer R (Neon Transfection System, ThermoFisher Scientific) containing 12 μg MIP-247 and 6 μg MIP-339. Cells were then electroporated (1400 V, 20 ms, 2 pulses) and placed into pre-warmed astrocyte media minus antibiotics + 10 μM Y-26732 on 1% Geltrex (ThermoFisher Scientific) coated 6-well plates (500k/well). The following morning the media was refreshed with astrocyte maintenance media + 10 μM Y-26732. On day 2, plates were washed with PBS and refreshed with reprogramming media 1 (DMEM/F12, P/S, 1× B27-Vitamin A, 1× Glutamax 20 ng/mL FGF2, 10 ng/mL FGF4, 10 ng/mL EGF) and refreshed bi-daily. On day 8, media was completely replaced by reprogramming media 2 (0.25 mM sodium butyrate, 50 μg/mL Ascorbic acid, 3 μM CHIR-99021, 0.5 μM A83-01, 10 μM Y-26732, 100 ng/mL FGF2). On day 13, cultures were supplemented with an additional 0.1 μM PD-0325901 and the base media was switched to 20% mTESR plus (STEMCELL Technologies): 80% media 2. Supplements remained at the same concentrations as media 2. On day 15, cells were moved to 50% mTESR plus: 50% media 2 and all reprogramming chemicals except Y-26732 were weaned by refreshing well volume by 50% daily with mTESR plus + 10 μM Y-26732. This was performed until day 20, this step was crucial to reduce cell death after treatment withdrawal. For TSC cells, rapamycin was added to the conversion at the noted time points to increase reprogramming efficiency (d1–d10, 20 nM; d11–d12, 10 nM; d13–d14, 1 nM). After d20 cells were maintained in iPSC media (mTESR plus, P/S) supplemented with 10 μM Y-26732 until clones were of sufficient size (300–1000 μm). After which they were excised with a 25-gauge needle under a stereo microscope. The selected colonies were seeded on 1% Geltrex (Gibco, Thermo Fisher Scientific, #A1413302) in 10 μM Y-26732, which was removed the following days. Cells were passaged using Accutase and centrifuged (250×*g*, 2′). To ensure pure iPSC cultures, cells were affinity-purified with TRA-1-60 beads at p5 (Anti-TRA-1-60 microbeads, Miltenyi Biotec, #130-100-832). iPSC lines were grown to p10 to establish a homogenous culture before downstream applications (Supplementary Fig. 1). When needed, cells were frozen down in Bambanker cryopreservative (Wako, #BB01) and thawed in the presence of 10 μM Y-26732 for 24 h. OCT4 expression was validated on protein level (Supplementary Fig. 2). Genomic integrity was confirmed at p10 by a human pluripotent stem cell (PSC) genetic analysis kit (Stemcell, #07550), mutational status by in-house sanger sequencing (Supplementary Fig. 3). Identity was confirmed by short tandem repeat (STR) analysis of tissue or parent line and the derived iPSC lines using the PowerPlex 16-system (Promega).

**Table 1:** Clinical data of generated lines

ID	PA Diag.	M/F	Onset age (Y)	Age (at op.)	Duration (Y)	Seizure freq. (M)	Area	TSC	Mutation type	HGVS annotation	Autism
*T1*	TSC	F	1	30	29	91	T	1	Stop-gain	c.163C>T	No
*T2*	TSC	F	1	17	16	200	F	2	Deletion	c.2525del	Yes
*T3*	TSC	M	0	2	2	304	F	2	Frameshift	c.4422_4423del	Yes
**T4**	TSC	M	0	4	4	120	F	2	Nonsense	c.1372C>T	No
**T5**	TSC	F	8	9	1	122	F	1	Frameshift	c.160dupT	No
**T6**	TSC	M	0	3	3	130	F	2	Exon deletion	14–41del	Yes
**T7**	TSC	M	0.3	3	2.7	60.8	F	2	Frameshift	c.827del	Yes

*F* frontal, *T* temporal, *Op* operation, *TSC* tuberous sclerosis complex, *PA Diag.* pathology diagnosis, *HGVS* human genome variation society

Bold underlined: used in functional experiments

#### iMGL-cells generation

Human iMGL-cells were generated using a previously published protocol [25, 85]*.* Briefly, embryonic bodies were formed in BVS-medium (mTSR+, P/S, 2 μM thiazovivin, 50 ng/mL BMP4, 50 ng/mL VEGF and 50 ng/mL SCF). Then the generation of hematopoietic cells is initiated by replating the EBs in SMIFT-medium (X-VIVO15, P/S, 1× Glutamax, 0.055 mM 2-mercaptoethanol, 50 ng/mL SCF, 50 ng/mL M-CSF, 50 ng/mL IL-3, 50 ng/mL FLT3 and 5 ng/mL TPO). For the final myeloid stage, SMIFT-medium was changed to FMG media (X-VIVO15, P/S, 1× Glutamax, 0.055 mM 2-mercaptoethanol, 50 ng/mL FLT3, 50 ng/mL M-CSF, 25 ng/mL GM-CSF). Harvested microglial progenitors were matured for 7–14 days at 50 k/cm^2^ in maturation media (advanced DMEM/F12 1:1, P/S, 1× B27, 0.5× N2, 5 μg/mL *N*-acetylcysteine, 1× GlutaMAX, 400 μM 1-thioglycerol, 1 μg/mL heparan sulfate, 0.5 × non-essential amino acids (NEAA), 100 ng/mL IL-34, 25 ng/mL M-CSF, 25 ng/mL CX3CL1, 25 ng/mL TGF-β and 50 ng/mL CD200) replenished every 4 days. To ensure consistency and minimize technical variation, all experiments were performed using iMGLs derived from the same differentiation batch, with progenitors harvested at the same stage and matured for the same period. The specific cell lines used in each experiment are listed in Supplementary Table 2.

#### Inflammatory response of iMGL-cells

On DIV 8, all medium was aspirated from mature iMGL cells and replaced with fresh microglial maturation medium supplemented with LPS (100 ng/mL) for 24 h. Control wells received fresh microglial maturation medium without LPS.

#### RNA isolation

Total RNA was extracted using Trizol reagent (Invitrogen). Cells were washed with phosphate-buffered saline (PBS), lysed, and subjected to phenol–chloroform extraction (1:0.14 ratio). Phase separation was achieved by centrifugation at 12,000×*g* for 15 min at 4 °C. The aqueous phase was carefully collected, mixed with an equal volume of isopropanol and 1 μL of glycogen-blue (Invitrogen), and precipitated overnight at − 20 °C. The following day, RNA was pelleted by centrifugation at > 20,000×*g* for 30 min at 4 °C and washed twice with ice-cold 75% ethanol. Pellets were air-dried at room temperature for 5 min, then resuspended in THE RNA solution (Invitrogen) supplemented with 20 mM DTT. Samples were incubated at 60 °C for 10 min to inactivate residual RNases.

#### cDNA synthesis and reverse-transcription quantitative PCR (RT-qPCR)

RNA concentration was measured using a Nanodrop spectrophotometer (ThermoFisher Scientific), and 250 ng of total RNA was used per reaction for cDNA synthesis using oligo-dT primers. Each RT-qPCR reaction consisted of 1 µL cDNA, 2.5 µL FastStart Reaction Mix SYBR Green I (Roche Applied Science), and 0.4 µM of both forward and reverse primers. The reaction volume was brought to a final total of 5 µL with RNase-free water. RT-qPCR was carried out on a LightCycler 480 system (Roche Applied Science), using small nuclear ribonucleoprotein D3 polypeptide (SNRPD3) as the reference gene. Data were analyzed using the LinRegPCR method as previously described [97]. For a detailed list of primers used see Supplementary Table 3.

#### Immunofluorescence of cultures

Cells were grown in 24-well plates on 13 mm HCl-etched coverslips (1 M HCl, 24 h at 60 °C, washed 3 times with H_2_0 and stored in 70% ethanol) and fixated with 4% paraformaldehyde for 15–20 min. Plates were gently washed with PBS 3 times and stored at 4 °C. Cells were permeabilized for 10 min at RT with 0.25% triton X-100 and blocked with 0.22 μm filtered 5% BSA in PBS + 0.1% tween-20 (PBST) for 1 h. After blocking, coverslips were incubated with 200 μL antibody solution (antibody + 1% BSA in PBST, see Supplementary Table 4 for antibodies and dilutions) overnight at 4 °C. Wells were washed 3 times with washing solution 1 (0.5% BSA, PBST) for 10 min on a gently tilting platform and subsequently incubated with secondary antibodies and phalloidin (actin) counterstaining in PBST. After secondary incubation, wells were washed 3 × 10 min with washing solution 2 (0.2% BSA in PBST) and counterstained with DAPI (0.5 μg/mL in PBS) for 5 min. After which, a final washing step (3× PBS, 10 m) was performed and coverslips were mounted on a microscope slide with ProLong Gold Antifade Mountant (Thermofisher, #P36930).

#### Protein isolation and immunoblotting

Cells were lysed on ice for 30 min in radioimmunoprecipitation assay buffer (RIPA) buffer (150 mM NaCl, 1% NP-40, 0.5% sodium deoxycholate, 0.1% SDS, 50 mM Tris pH 7.4) supplemented with 2× protease inhibitor cocktail (Merck) and 1× PhosSTOP (Roche). Lysates were sheared through a 25G needle, cleared by centrifugation (13,000×*g*, 10 min, 4 °C), and protein concentration was quantified using a BCA assay (ThermoFisher Scientific). Then, 15 µg of proteins were mixed with the right amount of Laemmli Sample Buffer 4X (Bio-Rad Laboratories), and boiled. The samples were then loaded onto a 4–20% Precast gel for SDS-PAGE (BioRad 4568094). The proteins were transferred onto a nitrocellulose membrane, using Mini Transfer Packs with Trans-Blot® Turbo TM (Bio-Rad Laboratories) according to the manufacturer’s instructions (Bio-Rad Laboratories). The membranes were blocked in 5% skimmed milk (Sigma, cat# 70166) for 45 min at room temperature. Subsequently, the membranes were incubated with the indicated primary antibody overnight at 4 °C. The primary antibodies used are: Anti-IP3R (Immunological Science AB-82747), Anti-SERCA2 (abcam ab255960), Anti-STIM 1 (Cell signaling, 4916), Anti-ORAI1 (Invitrogen PA-74181); Ponceau S Solution (Sigma P7170), and Anti-β-actin (Sigma-Aldrich A1978) were used to normalize the protein loading of IP3R, SERCA2, and STIM1, ORAI1, respectively. Goat anti-mouse IgG (H + L) horseradish peroxidase-conjugated (Bio-Rad, 1:5000; Cat. 170-6516) and Goat anti-rabbit IgG (H + L) horseradish peroxidase-conjugated secondary antibodies (Bio-Rad, 1:5000; Cat. 170-6515) were used. Detection was done with SuperSignalTM West Pico/femto PLUS Chemiluminescent Substrate (Thermo Scientific, Cat. 34578), based on the chemiluminescence of luminol, and developed using ChemiDocTM Imaging System (Bio-Rad). Western blot analysis of anti-POU5F1 (OCT3/4; Monosan, Cat. MONX11104) and anti-β-actin (Sigma, Cat. A5441) in iPSC samples was performed according to a previously described protocol [56].

### Measurement of intracellular Ca^2+^

Control and TSC iMGL cells were cultured on 24 mm round glass coverslips (2 × 10^5^ cells per coverslip) and loaded with 2.5 μM Fura-2 acetoxymethyl ester (Fura-2/AM; Life Technologies, Cat. No. F1201), in the presence of 0.005% Pluronic F-127 (Life Technologies, Cat. No. P6867) and 10 μM sulfinpyrazone (Sigma-Aldrich, Cat. No. S9509), prepared in Krebs–Ringer buffer (KRB: 125 mM NaCl, 5 mM KCl, 1 mM Na3PO4, 1 mM MgSO4, 5.5 mM glucose, 20 mM HEPES, pH 7.4) supplemented with 2 mM CaCl_2_.

Cells were incubated for 30 min in the dark to facilitate dye uptake, followed by a single wash with KRB and then incubated for an additional 30 min to allow de-esterification of the dye. Following preparation, coverslips were transferred to an acquisition chamber mounted on a Leica DM6000B epifluorescence microscope, equipped with a 40×/1.3 S Fluor objective. Fura-2 was alternately excited at 340 nm and 380 nm using a Polychrome V monochromator (Till Photonics, Munich, Germany), and fluorescence emission was collected through a 510/20 nm bandpass filter using a cooled CCD camera (Hamamatsu). Images were acquired and processed using MetaFluor software (Molecular Devices, Sunnyvale, CA, USA). To induce intracellular calcium signals, cells were stimulated with 50 μM ATP. For SOCE experiments, intracellular Ca^2^⁺ stores were first depleted by applying 50 μM *tert*-butylhydroquinone (TBHQ; Sigma-Aldrich) in a calcium-free external solution (basic KRB supplemented with 500 µM EGTA). SOCE was subsequently assessed by reintroducing 2 mM Ca^2^⁺ to the medium. Ca^2+^ imaging in the mitochondrial matrix was performed using a mitochondrially targeted variant of Fura-2, mt-fura-2.3 reported elsewhere [74], with the same procedure as for cytosolic Fura-2. For comparative analysis of calcium transients, changes were expressed as the normalized Fura ratio using the formula (*F*_*i*_ – *F*_*₀*_)/*F*_*₀*_, where *F*_*i*_ represents the current ratio at point (*i*) and *F*_*₀*_ is the baseline.

### High-resolution respirometry—OROBOROS

Real-time cellular respiration in control and TSC iMGL cells was assessed using the Oroboros Oxygraph-2k high-resolution respirometer (Oroboros Instruments, Innsbruck, Austria). The experiment followed the “substrate, uncoupler, inhibitor, titration” (SUIT) protocol, specifically SUIT-003_O2_ce_D012, as per the manufacturer’s recommendations and prior methodologies [79]. Cells were detached from 6-well plates using accutase and resuspended in pre-warmed MiR05 respiration buffer (composition: 0.5 mM EGTA, 3.0 mM MgCl_2_, 60 mM potassium lactobionate, 20 mM taurine, 10 mM KH_2_PO_4_, 20 mM HEPES, 110 mM sucrose, and 1 g/L BSA; pH 7.1). Both control and TSC-derived microglia were analyzed in parallel using adjacent respirometry chambers, with oxygen levels and fluxes monitored via DatLab software (Oroboros). Initial oxygen consumption rates were established under the “Routine” condition, both in the absence and presence of 5 mM pyruvate to support TCA cycle activity. Following this, 5 nM oligomycin was added to inhibit ATP synthase, allowing measurement of the “Leak” respiration. To evaluate the cells’ maximal respiratory capacity, the protonophore FCCP was titrated in 0.05 μM steps until peak oxygen consumption was observed (ET or “Electron Transport” phase). Subsequently, 1 μL each of rotenone (0.5 μM) and antimycin A (2.5 μM) were added sequentially to block mitochondrial complexes I and III, thereby measuring residual, non-ETC-related respiration (ROX phase). Oxygen flux values were normalized to total protein concentration. After the respirometry assay, cell suspensions from each chamber were centrifuged at 1000×*g* for 5 min. The resulting pellets were lysed in 200 µL of buffer (10 mM HEPES, 60 mM KCl, 1 mM EDTA, 0.075% NP-40, 1 mM DTT), and the lysates were centrifuged at 15,000×*g* for 15 min at 4 °C. Protein concentrations in the supernatant were determined using the Bradford assay.

### Crude synaptic fraction isolation and phagocytosis assay

Crude synaptic fractions were isolated from human fetal brain tissue (15–24 weeks gestation) using a modified Syn-PER protocol to improve purity [56]. Synaptosomes were labeled with pHrodo Red for fluorescence-based tracking, validated via pH titration and protein quantification [56]. For phagocytosis assays, cells were cultured in 96-well plates, and exposed to synaptosomes. Uptake was quantified over 24 h using IncuCyte S3 live-cell imaging. Image masks for the fluorescent signal were normalized to the cell body area [60].

### Statistical analysis

Statistical analyses were conducted using GraphPad Prism (GraphPad Software Inc., La Jolla, CA). Data normality was assessed using the Shapiro–Wilk test, and outliers were identified using the ROUT method (*Q* = 1%). Non-parametric comparisons between groups were performed using the Mann–Whitney *U* test. A *p*-value < 0.05 was considered statistically significant.

## Results

### Differential expression of genes related to Ca^2+^ signalling in TSC microglia from primary tissue

Traditionally, research on Ca^2^⁺ signalling in epilepsy has primarily focused on neurons, providing important insights into neuronal dysfunction. However, a more comprehensive understanding of epileptogenesis requires consideration of non-neuronal cell types. Recent studies investigating Ca^2^⁺ signalling in TSC-derived astrocytes from resected tubers have yielded novel insights into its interaction with mTOR hyperactivation [82]. While astrocytes have thus emerged as critical contributors, the role of microglia remains less well characterized. In these cells, neuronal activity rapidly triggers elevated Ca^2+^ signaling, which in turn drives diverse responses, including morphological changes, cytokine release and synaptic pruning [24, 54, 96]. Microglia are thus key players in neuroinflammation and network remodelling. Yet, in contrast to astrocytes, the dynamics of Ca^2^⁺ signaling in microglia have received comparatively little attention in epilepsy research. Exploring these processes may yield new perspectives on glial contributions to epileptogenesis in TSC and broaden our understanding of how altered Ca^2^⁺ homeostasis across multiple cell types shapes disease mechanisms.

**Fig. 1:**
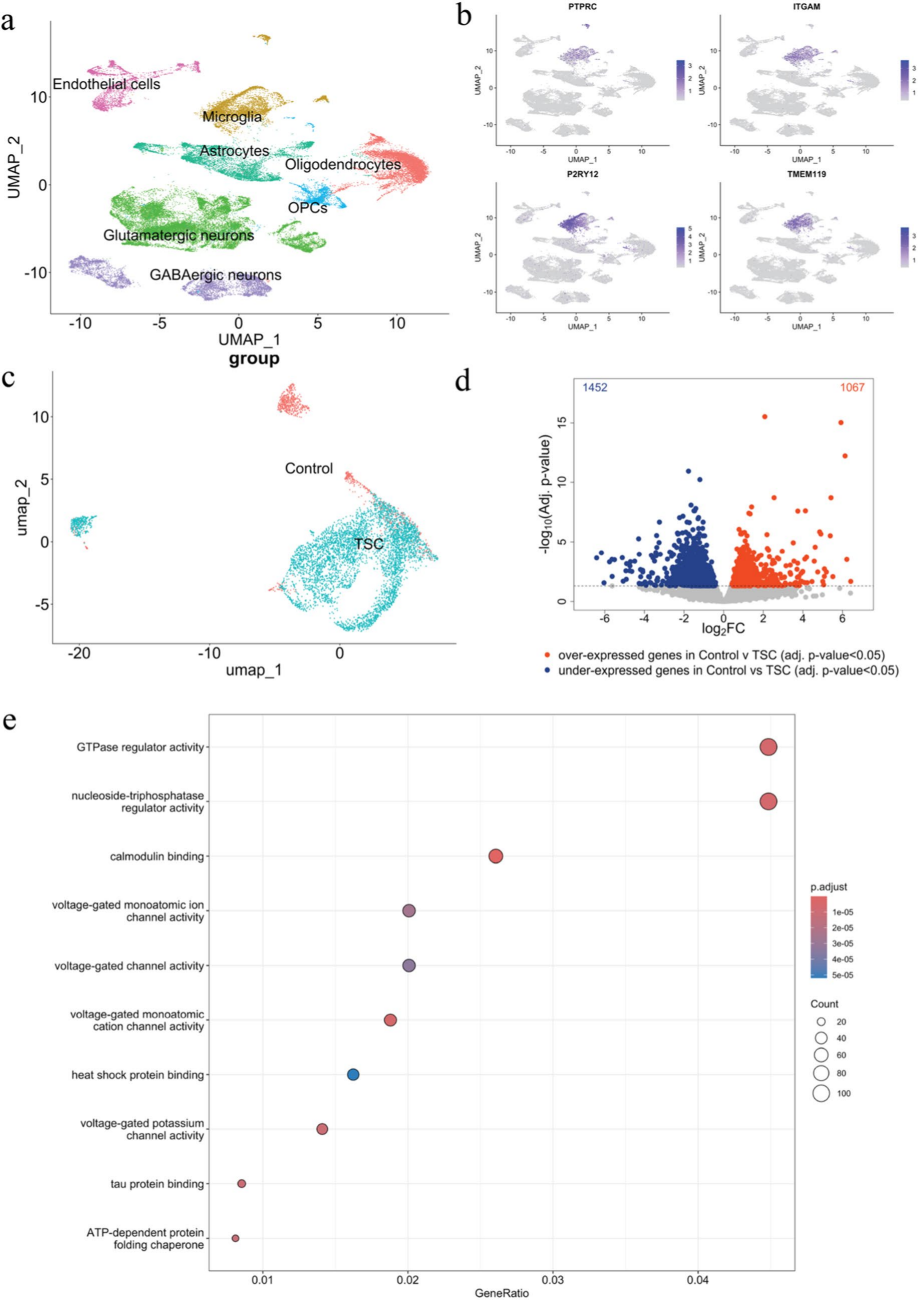
Single-cell RNA sequencing of control and TSC of primary tissue (cortical tubers). **a** Uniform Manifold Approximation and Projection (UMAP) plot displays the clustering of 7 cell types, including microglia. **b** The microglia-specific cluster was enriched for: protein tyrosine phosphatase receptor type C (*PTPRC*), Integrin alfa M (*ITGAM*), *P2YR12* and *TMEM119*. **c** UMAP displaying control and TSC-specific clustering of microglia. **d** Volcano plot showing the differential gene expression analysis, which is based on control (*n* = 3) and TSC (*n* = 5). **e** Gene Ontology (GO) molecular function enrichment of differentially expressed genes in TSC microglia. Bubble plot showing the top enriched GO molecular function terms among differentially expressed genes in microglia from TSC versus control cortex. The *x*-axis represents the GeneRatio (proportion of DEGs mapping to each GO term). Bubble size indicates the number of genes contributing to each term, and color reflects the adjusted *p*-value

**Fig. 2:**
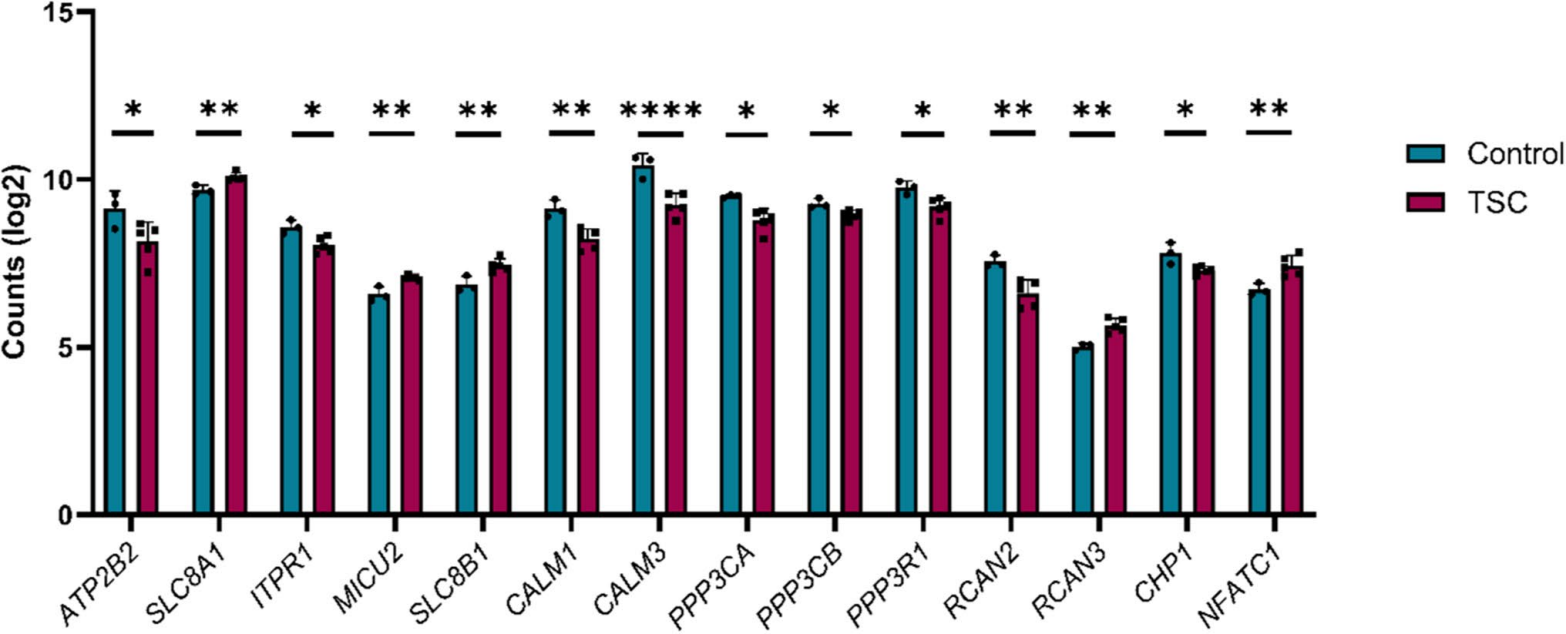
Differential expression of Ca^2^⁺-related genes reveal an alteration in TSC microglia using single-cell RNA sequencing on primary tissue (*n* = 3 control and *n* = 5 TSC). Statistical analysis was performed using DESeq2 with Benjamini–Hochberg correction for multiple testing. Data are expressed as mean ± SEM. Adjusted *p*-values indicated by * for *p* < 0.05, ** for *p* < 0.01, ****p < 0.0001

To explore this concept, we analyzed cortical brain tissue resected from patients with pharmacoresistant epilepsy diagnosed with TSC cortical tubers (*n* = 5), alongside age- and region-matched autopsy control samples (*n* = 3) from individuals without a history of seizures or neurological disease (Supplementary Table 1). Using scRNA-seq, we identified 10 distinct microglia populations in our cortical brain samples in the control cohort and 9 in the TSC cohort. To investigate Ca^2^⁺-related transcriptional dysregulation specifically within microglia, we first isolated all clusters annotated as microglia from the integrated dataset. The extracted cells exhibited strong enrichment for canonical microglial markers, including protein tyrosine phosphatase receptor type C (*PTPRC*), Integrin alfa M (*ITGAM)*, *P2YR12* and *TMEM119* (Fig. 1a–c), confirming accurate cell-type identity. By comparative analysis of control (*n* = 3) and TSC (*n* = 5) tissue, we identified more than 2000 differentially expressed genes (DEGs) between control and TSC microglia (Figs. 1d, 2). A summary of the top 15 up- and down-regulated genes is provided in Supplementary Table 5.

Following the differential expression analysis, we performed an unbiased Gene Ontology (GO) enrichment analysis on microglial DEGs to identify overrepresented molecular functions. This analysis revealed significant enrichment for pathways related to ion channel activity, including “voltage-gated channel activity” and “voltage-gated monoatomic ion/cation channel activity”, indicating broad changes in microglial electrophysiological and membrane signalling properties (Fig. 1e). In addition, terms associated with protein binding and stress responses, such as “heat shock protein binding” and “ATP-dependent protein folding chaperone”, were enriched, suggesting altered proteostasis in TSC microglia. Together, these findings indicate that microglial transcriptional alterations in TSC extend beyond inflammatory programs and include shifts in ion-channel-related and signalling pathways, providing a rationale for subsequently examining calcium-associated mechanisms in greater detail.

Next, we examined the expression profiles of 62 expertly curated genes implicated in Ca^2^⁺ signalling, of which 14 of 62 were differentially expressed, representing a significant enrichment (hypergeometric test; *p* < 0.05).

We found that the following genes were downregulated in TSC: *ATP2B2* (Log_2_FC = − 1.426; *p*.adj = 0.035), *ITPR1* (Log_2_FC = − 0.827; *p*.adj = 0.025), *CALM1* (Log_2_FC = − 1.354; *p*.adj = 0.001), *CALM3* (Log_2_FC = − 1.741; *p*.adj = 0.0001), *PPP3CA* (Log_2_FC = − 1.27; *p*.adj = 0.010); *PPP3CB* (Log_2_FC = − 0.517; *p*.adj = 0.049), *PPP3R1* (Log_2_FC = − 0.843; *p*.adj = 0.023); *RCAN2* (Log_2_FC = − 1.443; *p*.adj = 0.004); *RCAN3* (Log_2_FC = 1.361; *p*.adj = 0.005), *CHP1* (Log_2_FC = − 0.853; *p*.adj = 0.010), while following genes were upregulated in TSC microglia, *SLC8A1* (Log_2_FC = 0,61; *p*.adj = 0.01), *MICU2* (Log_2_FC = 0.85; *p*.adj = 0,005), *SLC8B1* (Log_2_FC = 0.97; *p*.adj = 0.009), *NFATC1* (Log_2_FC = 1.215; *p*.adj = 0.006).

Beyond calcium signalling, we also detected transcriptional features indicative of a change in microglial activation state. Several genes previously associated with stage 2 DAM programs, including *TREM2*, *CLEC7A*, *LPL*, *AXL*, *ITGAX*, and *CSF1*, were upregulated in TSC microglia (Supplementary Fig. 4). This suggests that TSC microglia may partially adopt DAM-like features, indicating a shift toward an activated state, beyond homeostatic microglial function.

These results, suggesting global changes in Ca^2^⁺ homeostasis and signalling at a cellular resolution, and a shift towards a hyperactive phenotype, prompted us to investigate in depth functional alterations in TSC microglial Ca^2^⁺ signalling and microglia-specific Ca^2^⁺-regulated functions, such as phagocytosis.

## Generation of iPSC from TSC patient astrocytes and derivation into iMGL cells

**Fig. 3:**
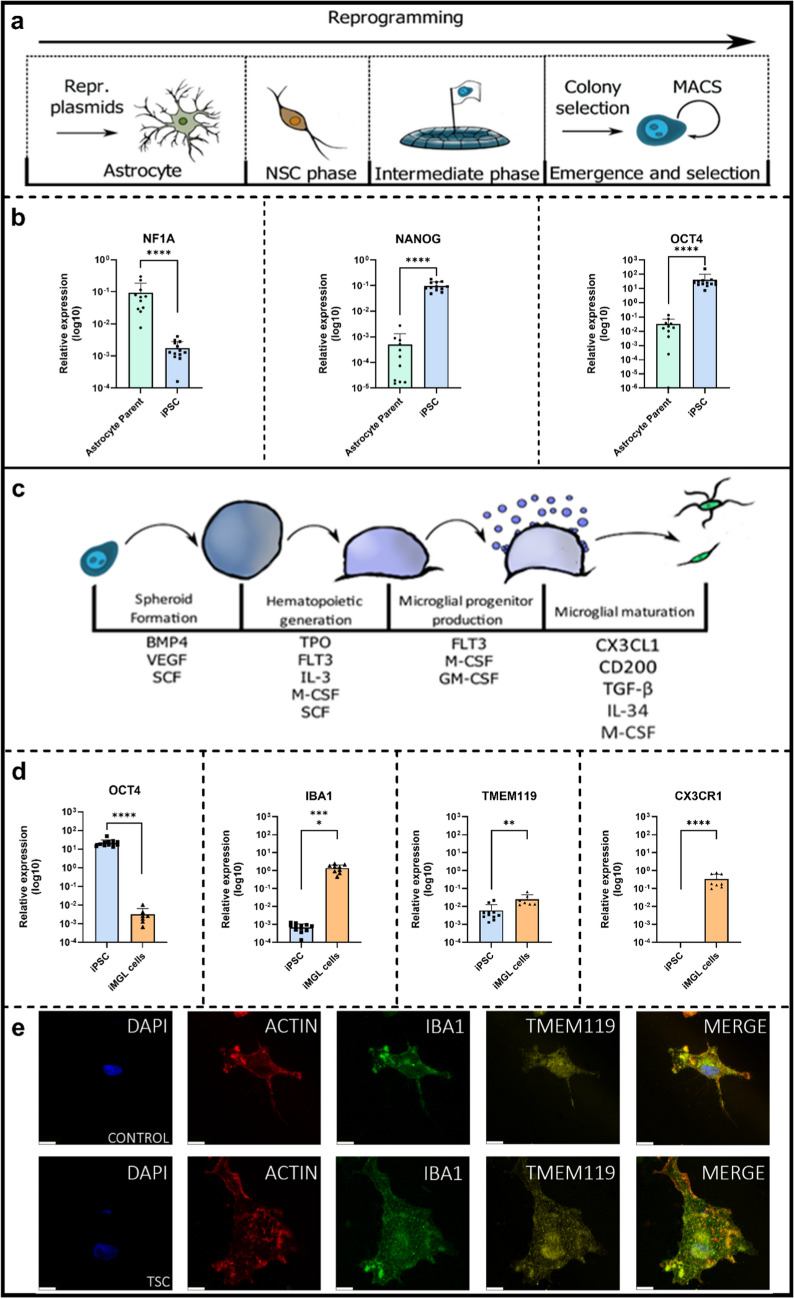
Overview of astrocyte reprogramming to iPSCs and differentiation into iMGL cells. **a** Schematic overview of astrocyte-to-iPSC reprogramming. **b** Primary astrocytes are reprogrammed into iPSCs, characterized by the downregulation of astrocytic lineage marker *NFIA* and concurrent upregulation of pluripotency markers *NANOG* and *OCT4 (n* = 11 astrocyte parents, *n* = 12 iPSCs). **c** Schematic overview of stepwise differentiation of iPSCs into iMGL cells. **d** iPSCs are differentiated into iMGL cells, characterized by the downregulation of pluripotency markers (*OCT4*) and the upregulation of microglial markers (*IBA1, TMEM119, CX3CR1*) (*n* = 12 iPSCs, *n* = 9 iMGL cells). **e** Immunofluorescence (IF) staining confirms the identity of control and TSC-iMGL cells, showing robust expression of IBA1, and the microglia-specific marker, TMEM119. Representative IF images display co-localization of these markers (Scale bar: 10 µM). *NSC* neural stem cell, *MACS* magnetic activated cell sorting, *iMGL* iPSC-induced microglia like. *p*-values indicated by ** for *p* < 0.01, **** for *p* < 0.0001. Mann–Whitney *U* test was used to test significance

To investigate how alterations in intracellular Ca^2^⁺ dynamics and microglial function are affected, we employed an iMGL cells model, as previously described [25, 85]. iPSC lines were generated by reprogramming human astrocytes isolated from either cortical tubers of TSC patients (*n* = 7, see Table 1) or fetal brain tissue (*n* = 5) (Fig. 3a). Reprogramming was achieved using episomal plasmid transfection [22], with detailed media compositions and steps provided in the methods section. Pluripotency was validated by assessing the loss of the glial transcription factor *NF1A* [93] and the expression of key iPSC markers, including *NANOG* and *OCT4* (Fig. 3b). OCT4 expression was further confirmed at the protein level (see Supplementary Fig. 2). Additional quality control data for iPSC generation are presented in Supplementary Fig. 3.

Subsequently, we differentiated iPSCs into iMGL cells using an established embryoid body-based protocol (Fig. 3c) [25, 85]. Microglial identity was validated with the downregulation of pluripotency marker *OCT4*, and the upregulation of microglial markers *IBA1*, *TMEM119*, *CX3CR1* (Fig. 3d). Immunofluorescence staining of the iMGL cells confirmed their expected morphology, as well as the expression of microglial markers IBA1 and TMEM119 (Fig. 3e). Upon 24 h LPS stimulation, iMGL cells mounted a clear inflammatory response, characterized by increased expression of pro-inflammatory markers, IL-6, IL-1β, TNFα, and anti-inflammatory marker IL-10, consistent with functional microglial activation (Supplementary Fig. 5). Under non-stimulated conditions, TSC iMGL cells exhibited reduced TNFα and IL-10 expression compared with controls. While the difference in IL-10 expression was no longer observed after LPS stimulation, TNFα levels remained lower in TSC iMGL cells. This suggests that TSC IMGL cells may have an altered, potentially attenuated inflammatory response, a finding consistent with previous in vivo studies [88, 107].

## Exaggerated cytosolic Ca^2+^ signaling in response to ATP in TSC iMGL cells

**Fig. 4:**
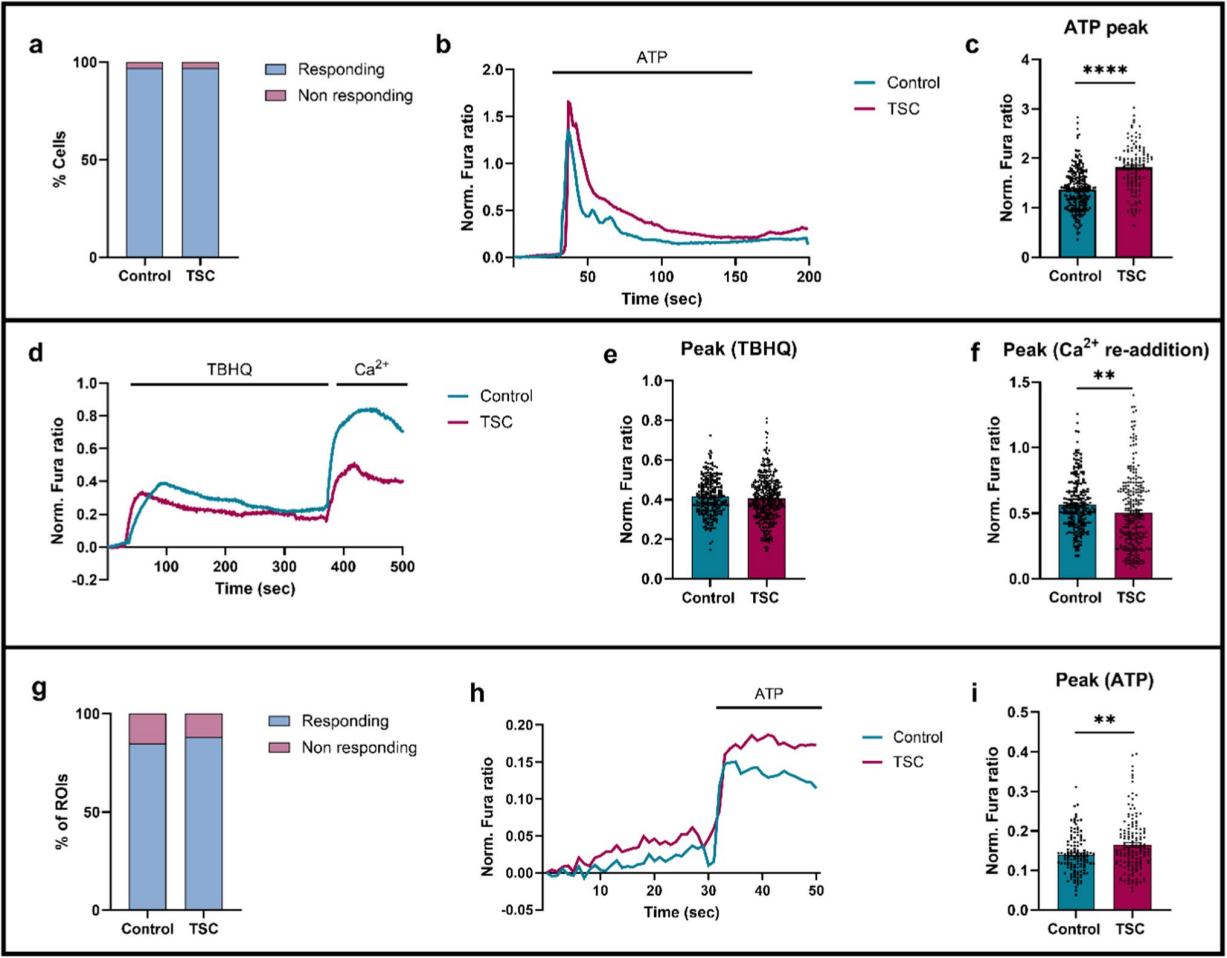
Ca^2+^ imaging in the cytosol and mitochondria of control and TSC iMGL cells. **a** The percentage responding versus non-responding cells was similar between the control and TSC group (light blue: responding; pink: non-responding cells). **b** Both iMGL cells were loaded with Fura-2/AM and challenged with 50 µM ATP in Ca^2+^ containing KRB solution. Representative curve of cytosolic Ca^2+^ response in both groups. **c** After stimulating the cells with ATP, the amplitude of the cytosolic Ca^2+^ response was higher in TSC iMGL cells compared to control iMGL cells (*n* = 257 control cells and *n* = 120 TSC cells, from 11 independent coverslips). **d** Control and TSC iMGL cells were loaded with Fura-2/AM and consecutively stimulated with TBHQ (after 50 s) and Ca^2^⁺ (after 380 s). Representative traces of cytosolic Ca^2+^ response in both groups. **e** After stimulating with TBHQ, which blocks SERCA, no differences of Ca^2^⁺ concentration in the cytoplasm was observed between both groups. **f** After re-addition of Ca^2^⁺ (2 mM) we observed a reduction of Ca^2^⁺ concentration in the ER in TSC iMGL cells (*n* = 231 control cells and *n* = 326 TSC cells, from 17 independent coverslips). **g** No difference between the percentage of cells showing mitochondrial Ca^2+^ response were observed between both groups. **h** Cells were loaded with mt-fura-2.3 and challenged with ATP. Representative trace of mitochondrial Ca^2+^ response in control and TSC-IPSC-derived microglia. After 30 s, cells were stimulated with 50 µM ATP. **i** After stimulation with ATP, we observed an increase in mitochondrial Ca^2+^ response in TSC iMGL cells compared to control (*n* = 124 control ROIs and *n* = 148 TSC ROIs, from 11 independent coverslips). Data shown as mean ± SEM for bar graphs. *p*-values indicated by ** for *p* < 0.01 and **** for *p* < 0.0001. Mann–Whitney *U* test was used to test significance

After patient transcriptomic analysis and iPSC/iMGL cell generation, we investigated whether Ca^2+^ handling is altered in TSC iMGL cells compared with healthy controls. In their resting, ‘surveillant’ state, microglial Ca^2^⁺ signaling remains minimal within a healthy brain environment [29]. In a pathological context, damage-associated-molecular patterns (DAMPs), including ATP and ADP, can activate microglia, thereby generating Ca^2+^ signals [29, 36, 44]. To challenge our iMGL cells, and elicit a Ca^2+^ response, we stimulated the cells with ATP (50 µM). While no differences were observed in basal cytosolic Ca^2+^ levels, ATP addition induced a rapid and robust increase of cytosolic Ca^2+^ in Fura-2 loaded iMGL cells, followed by a progressive decrease and re-establishment of near-basal Ca^2+^ levels within approximately 100 s after stimulation. The proportion of responding cells was comparable between the two groups (Fig. 4a). However, ATP stimulation resulted in a significantly greater increase in cytosolic Ca^2^⁺ level [Ca^2^⁺]c in TSC iMGL cells, as demonstrated by the enhanced peak amplitude and distinct curve profiles (Fig. 4b,c), suggesting enhanced responsiveness of TSC iMGL cells to Ca^2+^-ATP.

## SOCE impairment in TSC iMGL cells

Under resting conditions, the ER continuously loses Ca^2^⁺ through passive leakage, a process counteracted by SERCA, which replenishes ER Ca^2^⁺ stores [26, 50, 76]. In TSC, hyperactivation of mTOR signaling disrupts this delicate balance by inducing ER stress, potentially contributing to Ca^2^⁺ dysregulation [20]. Furthermore, mTOR hyperactivation leads to the generation of reactive oxygen species (ROS), which further exacerbate ER dysfunction and cellular stress [20]. Enhanced ATP-induced Ca^2^⁺ mobilization in TSC microglia may result from increased Ca^2+^ storage in the ER. Moreover, depletion of ER Ca^2+^ stores during stimulated Ca^2+^ release, triggers Ca^2+^ entry from the extracellular space via SOCE. In microglial cells, SOCE is required not only for the replenishment of the ER Ca^2+^ pool but also for activation of microglia-specific processes such as secretion of cytokines, cellular motility and immune responses [29].

To investigate whether ER Ca^2+^ storage and SOCE are altered in TSC iMGL cells, we applied a classical protocol consisting of ER Ca^2+^ depletion followed by Ca^2+^ re-addition. To achieve complete ER Ca^2^⁺ depletion, we applied TBHQ, a SERCA inhibitor, in Ca^2+^-free conditions. TBHQ induces accumulation of Ca^2^⁺ in the cytosol by preventing ER Ca^2^⁺ reuptake, leading to depletion of ER Ca^2^⁺ stores. To assess SOCE capacity, extracellular Ca^2^⁺ (2 mM) was subsequently reintroduced. Intracellular Ca^2^⁺ levels were measured using the ratiometric dye Fura-2 in a Ca^2^⁺-free KRB. After 30 s, cells were treated with 50 μM TBHQ, and after 300 s, extracellular Ca^2^⁺ was reintroduced (Fig. 4d).

Following TBHQ treatment, no significant differences were observed in cytosolic Ca^2+^ accumulation suggesting that the ER Ca^2^⁺ storage in TSC iMGL cells is unaffected (Fig. 4e). However, upon re-addition of extracellular Ca^2^⁺, TSC iMGL cells exhibited a marked reduction in Ca^2^⁺ influx compared to controls (Fig. 4f), suggesting impairment of SOCE.

## Increased mitochondrial Ca^2+^-signaling in response to ATP

Our findings demonstrate that extracellular ATP stimulation induces a rapid increase in [Ca^2+^]c. Mitochondria are highly responsive to cytosolic Ca^2+^ changes, and upon sensing elevated [Ca^2+^]c, Ca^2+^ enters the mitochondria via the MCU [34, 99]. In the present study, mitochondrial Ca^2+^ concentration [Ca^2+^]_m_ was measured with a variant of Fura-2 targeted to the mitochondrial matrix, mt-fura-2.3 [74].

After addition of ATP (50 µM), the proportion of responding mitochondria was comparable between the two groups (Fig. 4g). Upon ATP stimulation, we observed a greater increase of [Ca^2+^]_m_ in TSC iMGL cells compared to control (Fig. 4h, i). In the mitochondrial matrix, [Ca^2+^]_m_ is tightly linked to the activity of tricarboxylic acid (TCA) enzymes, regulating both the cycle itself and overall metabolic output [38]. An increase of [Ca^2+^]_m_ can, therefore, potentially enhance TCA-enzyme activity, which may lead to elevated mitochondrial respiration and ATP production [31, 38, 81].

Taken together, assessment of Ca^2+^ handling in TSC iMGL cells suggests enhanced stimulation-induced Ca^2+^ signals in the cytosol and mitochondria and a reduction of Ca^2+^ influx via the SOCE mechanism when assessed using the depletion-readdition protocol.

## Molecular alterations of Ca^2+^-related genes and proteins in iMGL cells

Given that ER Ca^2+^ levels did not change in TSC iMGL cells compared with control iMGL cells, we hypothesized that enhanced ATP-induced cytosolic Ca^2+^ signals could be due to altered expression of Ca^2+^ release channels in the ER membrane. Likewise, enhanced Ca^2+^ entry upon re-addition of Ca^2+^ in the SOCE experiment may be due to altered expression of SOCE-related molecules, STIM and ORAI. To investigate possible molecular determinants of functional Ca^2+^ signaling alterations in TSC iMGL cells, we used a combination of RT-qPCR and Western blot analyses of selected targets.

**Fig. 5:**
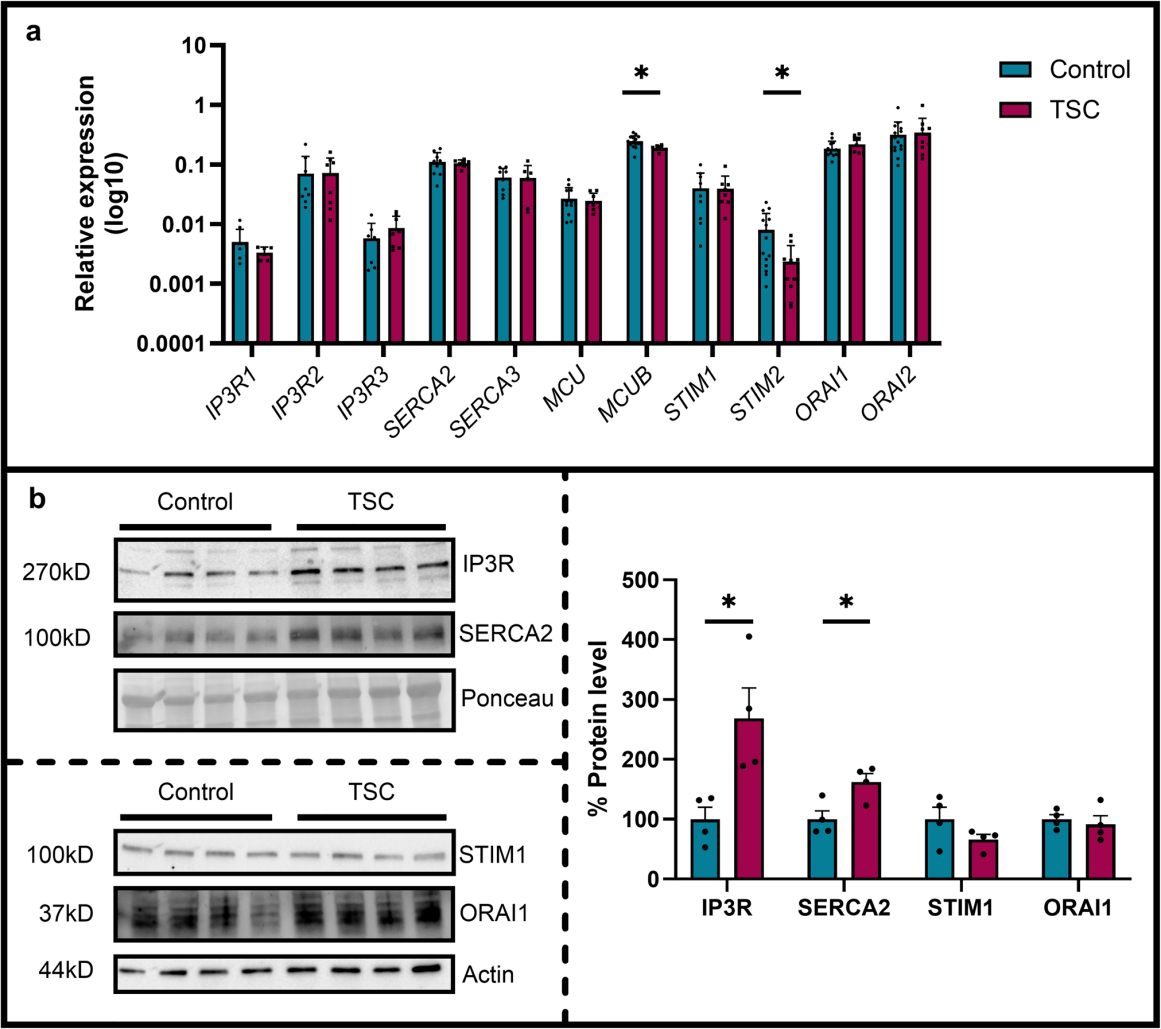
Alterations in Ca^2+^ related genes and protein expression in TSC iMGL cells. **a** RT-qPCR of Ca^2+^ channels (*IP3R1, IP3R2* and *IP3R3*) and of genes involved in general Ca^2+^ homeostasis (*SERCA2, SERCA3*), mitochondrial Ca^2+^ homeostasis (*MCU, MCUB*), SOCE (*STIM1, STIM2, ORAI1, and ORAI2*), normalized to the *SNRPD3* housekeeping gene (*n* = 4 control and *n* = 3 TSC, from ≥ 2 independent experiments). **b** Western blot analysis of selected Ca^2+^ related genes, IP3R and SERCA2 normalized on Ponceau, STIM1 and ORAI1 on Actin (*n* = 4 control and *n* = 4 TSC). Data shown as mean with SEM for bar graphs. *p*-values indicated by * for *p* < 0.05, ** for *p* < 0.01

RT-qPCR analysis was used to compare the levels of transcripts of intracellular Ca^2+^ release channels *IP3R1-3, SERCA2,3* pump, genes related to mitochondrial Ca^2+^ uptake *MCU* and *MCUB* and SOCE-related molecules *STIM1,2* and *ORAI1,2*. Of these, *MCUB* and *STIM2* were significantly down-regulated (*p* < 0.05, Fig. 5a), while other transcripts did not significantly change. Western blot analysis revealed a dramatic ≈170% upregulation of IP3Rs (*p* < 0.05, Fig. 5b, as detected by anti-pan-IP3R antibody, while SERCA2 pump was upregulated by about 60% (*p* < 0.05, Fig. 5b).

These results suggest that enhanced ATP-induced cytosolic Ca^2+^ signals in TSC iMGL cells may be due to the upregulation of IP3-sensitive Ca^2+^ channels on the plasma membrane. Furthermore, alongside the increased ER Ca^2+^ release, down-regulation of *MCUB*, a mitochondrial uniporter channel subunit exerting a dominant-negative effect on Ca^2+^ uptake [77], may contribute to the enhanced mitochondrial matrix Ca^2+^ signals in TSC iMGL cells. Regarding SOCE, we observed a significant reduction of *STIM2* transcript level and a similar trend toward reduced STIM1 protein expression, while ORAI1 expression remained unchanged at both mRNA and protein level. Although the mRNA and protein abundance did not correlate directly, these findings suggest that altered STIM expression, rather than ORAI abundance, may contribute to the modulation of SOCE in TSC iMGL cells.

Overall, the results of molecular analysis are consistent with functional alterations of Ca^2+^ homeostasis: enhanced agonist-stimulated cytosolic and mitochondrial Ca^2+^ signals and reduced SOCE. Moreover, these results suggest a differential regulation (transcriptional vs translational) of key components of the Ca^2+^ signaling downstream of over-activated mTOR in TSC iMGL cells.

## Increased oxygen consumption and maximum respiratory capacity in TSC iMGL cells

Upon ATP stimulation, we observed a greater increase of [Ca^2+^]_m_ in TSC iMGL cells compared to control (Fig. 4h, i). In the mitochondrial matrix, [Ca^2+^]_m_ is tightly linked to the activity of tricarboxylic acid (TCA) enzymes, regulating both the cycle itself and overall metabolic output [38]. An increase of [Ca^2+^]_m_ can, therefore, potentially enhance TCA-enzyme activity, which may lead to elevated mitochondrial respiration and ATP production [31, 38, 81].

**Fig. 6:**
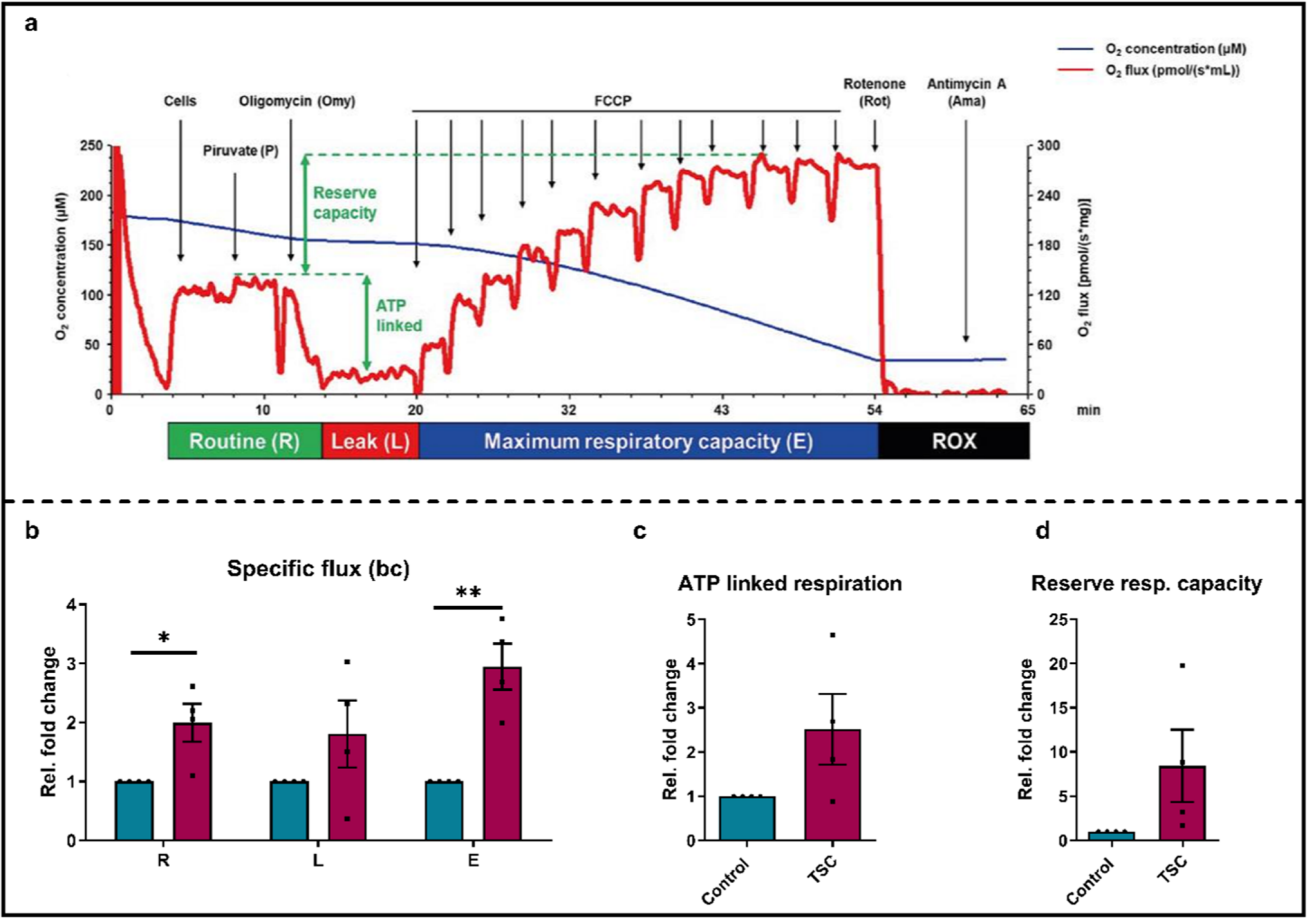
Oxygen consumption rate of control and TSC-iPSC-derived microglia normalized to protein content. **a** Representative trace of the distinct phases of the OROBOROS. **b** Oxygen flux in the routine state (R); leak state (L); and the maximum respiratory capacity (E). **c** ATP-linked respiration, obtained by the subtraction of L from R. **d** Reserve respiratory capacity obtained by the subtraction of R from E. Data shown as mean ± SEM as fold change to control (*n* = 4). *p*-values indicated by * *p* < 0.05, ** for *p* < 0.01. Mann–Whitney *U* test was used to test significance

To test this hypothesis, we assessed mitochondrial respiratory activity using the OROBOROS oxygraphy technology (Fig. 6a). The results of the OROBOROS analysis indicated that TSC iMGL cells exhibited an increase in two of the three distinct phases of the electron transport chain (ETC, Fig. 6b): basal oxygen consumption (R phase, *p* < 0.05) and maximum capacity (E phase, *p* < 0.01). The increase in basal oxygen consumption and maximum respiratory capacity of TSC iMGL cells indicates a higher energy demand compared to control. No differences were observed between ATP-linked respiration (*p* = 0.11) and reserve respiratory capacity (*p* = 0.12), albeit there seems to be a trend towards an increase in TSC iMGL cells (Fig. 6c, d).

The observed increase in basal oxygen consumption, maximum respiratory capacity and a trend towards an increase in ATP-linked respiration and reserve respiratory capacity, suggests a hypermetabolic phenotype of TSC iMGL cells when compared to controls.

## TSC iMGL cells exhibit increased phagocytic activity

**Fig. 7:**
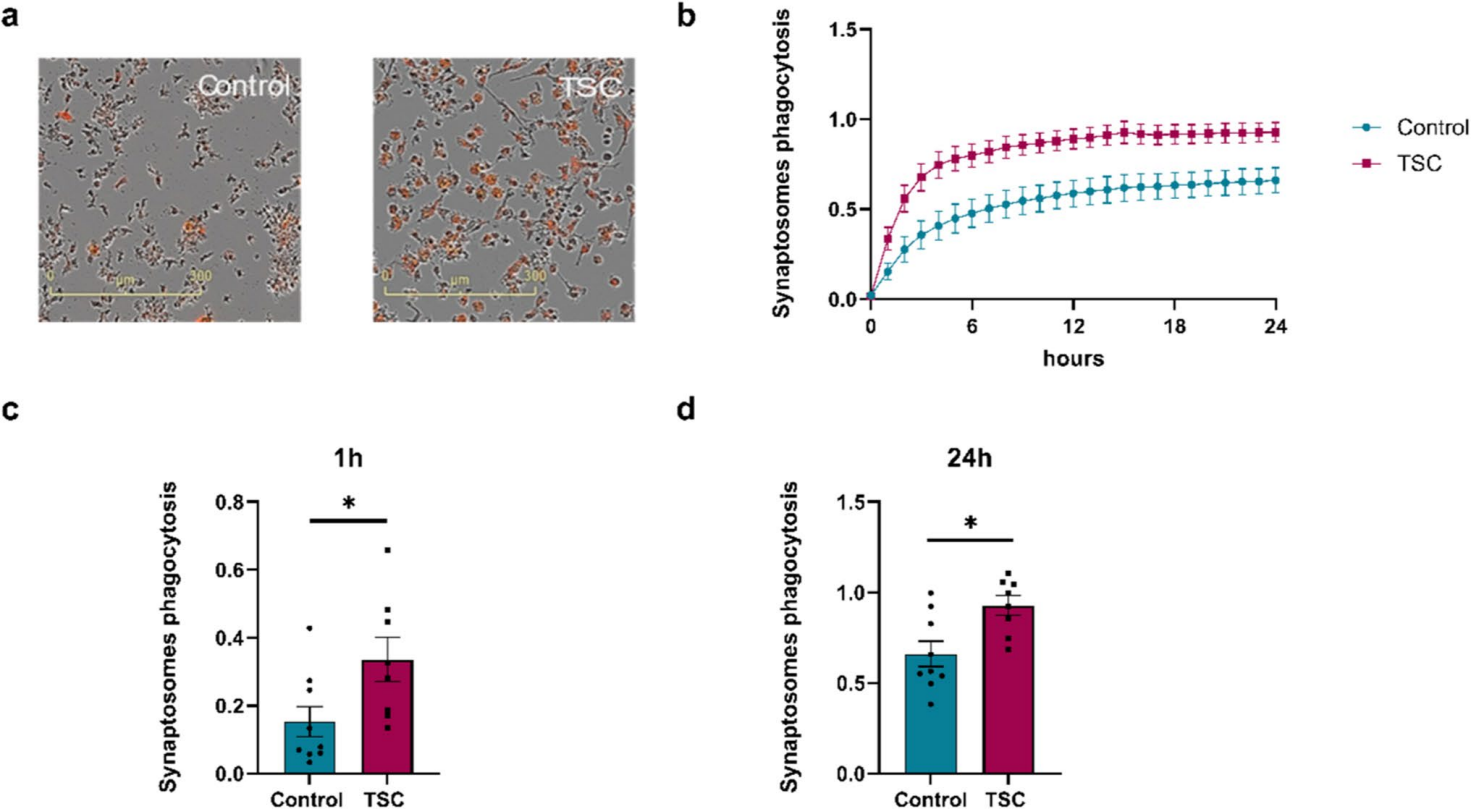
Increased phagocytic activity of TSC iMGL cells. **a** Representative images at 24 h with the IncuCyte S3 live imaging system. Human synaptosomes were labelled with Phrodo-red Scale bar: 300 µm. **b** After the addition of human synaptosomes, phagocytic activity was tracked for 24 h. **c**, **d** After 1 h and 24 h, TSC iMGL cells showed increased phagocytic activity (*n* = 4 control and *n* = 3 TSC, from ≥ 2 independent experiments). Data shown as mean ± SEM as fold change to control. *p*-values indicated by * *p* < 0.05. Mann–Whitney *U* tests were used to test significance

Ca^2+^ signalling in microglia has been linked to their phagocytic function, occurring either prior or during phagocytosis [24, 37, 46, 66, 96]. The observed increase in Ca^2+^ signalling and elevated metabolic state of the TSC iMGL cells led us to hypothesize that their phagocytic capacity might also be altered. In order to investigate this capacity, we challenged the iMGL cells with pHrodo-labeled human synaptosome fractions for a 24 h period (Fig. 7a, b). We discovered that TSC iMGL cells exhibit increased phagocytosis of synaptosomes in an acute (1 h) and chronic (24 h) time frame (Fig. 7c, d, *p* < 0.05).

## Discussion

In the present study, we broadened our understanding of Ca^2+^ signalling, intracellular dynamics, metabolic profile and phagocytic capacity of TSC iMGL cells. To investigate these aspects, we first analysed the transcriptomic profiles of resected TSC brain tissue. Based on these sequencing results, we employed an iMGL cell model to explore the consequences of the alterations on Ca^2+^ dynamics and respiratory capacity [25, 85]. Guided by these insights, we then examined microglial phagocytic activity. Collectively, our results provide new insights into the interplay between Ca^2^⁺ signaling, metabolic dynamics, and phagocytic function, supporting the emergence of a hypermetabolic, hyperresponsive, potentially stage 2 DAM-like phenotype, as corroborated by transcriptomic analysis of TSC brain tissue.

Microglia are the resident immune cells of the brain that mediate innate immune responses and inflammation, particularly during events such as CNS infections or traumatic brain injuries [19, 32, 44]. Beyond their immune role, microglia are essential for brain development and maintenance, regulating processes such as synaptic pruning and plasticity [71]. Under certain pathological conditions, however, microglia activation may become maladaptive, contributing to neuronal dysfunction and epileptogenesis [19, 21]. This phenotype is frequently observed in both TSC patients and preclinical models, where increased microglial activation and density have been described [6, 7, 41, 45, 88, 105, 106, 109]. Microglial activation is a key factor in epilepsy and associated comorbidities, correlating in TSC with white matter pathology and cognitive deficits [1, 21]. Inflammatory mediators further modulate ion channels and receptors, including GABAA, affecting the excitation/inhibition balance [83]. Chronic immune dysregulation is reinforced by impaired resolution mechanisms and epigenetic changes, such as IL1β promotor hypomethyliation, while transcriptomic profiling reveals links to oxidative stress, mitochondrial dysfunction, and prolong inflammatory signaling [2, 8, 28, 63]

In microglia, elevated Ca^2+^ signaling is rapidly triggered by neuronal activity and drives a range of responses, including morphological changes and pro-inflammatory cytokine release [24, 54, 96]. The elevated activation state observed in TSC microglia suggests that microglial Ca^2+^ dynamics might also be altered. Supporting this hypothesis, differential gene expression data of TSC brain tissue revealed dysregulations in genes associated with general Ca^2+^ homeostasis (*ATP2B2, SLC8A1*), mitochondrial Ca^2+^ homeostasis (*MICU2, SLC8B1*), Ca^2+^ buffering and signaling (*CALM1, CALM3, PPP3CA, PPP3CB, PPP3R1, RCAN2, RCAN3; CHP1*), and Ca^2+^ regulated inflammation (*NFATC1*). Although some of these alterations were not fully recapitulated in our iMGL model, we observed dysregulations of key Ca^2+^ related genes at both RNA (*MCUB, STIM2*) and protein (IP3R, SERCA2) levels. Discrepancies between tissue and culture likely reflect the complexity of the in vivo environment, where extensive crosstalk occurs between neurons and glia (Fig. 1). Given the high sensitivity of microglia to external cues, such as neuronal activity, which enhances calcium dynamics, it is therefore likely that certain changes present in vivo may not be fully captured in vitro. While these abnormalities may manifest differently in monoculture compared to the TSC brain, their presence supports the conclusion that TSC microglia harbor cell-intrinsic alterations in Ca^2+^ signalling that may contribute to aspects of the disease pathophysiology.

Functionally, TSC iMGL cells exhibit profound alterations in Ca^2+^ signaling, mitochondrial activity, and effector behaviors. Notably, we observed elevated cytosolic and mitochondrial Ca^2+^, following ATP stimulation, despite impaired SOCE and intact ER stores. In microglia, elevated cytosolic Ca^2+^ levels following ATP stimulation are typically driven by ER Ca^2+^ release through IP3 receptors, followed by sustained Ca^2+^ influx via SOCE [44]. In our TSC iMGL cells, ER Ca^2+^ remains intact, suggesting that this IP3-mediated release is preserved, while upregulation of IP3R explains enhanced cytosolic Ca^2+^ signals. However, we observed an impairment of SOCE, likely due to the downregulation of *STIM2*, a key ER Ca^2+^ sensor that gates ORAI1-mediated Ca^2+^ influx [4, 9, 62].

The concomitant rise in mitochondrial Ca^2+^ may be attributed to the downregulation of *MCUB*, a negative regulator of MCU [33, 72, 77, 78]. Under stress conditions, MCUB expression is typically upregulated, reducing the risks of mitochondrial Ca^2+^ overload by inhibiting Ca^2+^ uptake [48, 49, 78]. Thus, it is likely that loss of MCUB could lead to increased Ca^2+^ uptake by MCU beyond normal levels. Mitochondrial Ca^2+^ levels are tightly linked to the activation of several TCA-cycle enzymes, including pyruvate dehydrogenase, isocitrate dehydrogenase and α-ketoglutarate dehydrogenase [18, 31, 81], thereby enhancing NADH production and ETC activity. This mechanism may contribute to the increase in basal oxygen consumption and maximum respiratory capacity, suggesting a hyperactive phenotype for TSC iMGL cells [34]. Furthermore, the hyperresponsive and hypermetabolic state of TSC iMGLs is associated with an altered inflammatory response, suggesting that functional remodeling of neural networks by these cells could also occur independently of classical cytokine-driven inflammation. Interestingly, this hyperactive microglial phenotype contrasts with primary TSC astrocytes, where we recently reported reduced Ca^2^⁺ signalling and impaired mitochondrial metabolism [82]. Astrocytes are key homeostatic cells in the CNS, and proper Ca^2^⁺ signalling is critical for maintaining neuronal excitability, supporting synaptic transmission, and coordinating neuronal networks [53, 67, 92]. Although the precise molecular alterations across astrocytic subpopulations in TSC remain insufficiently characterized, evidence suggests that TSC astrocytes fail to fully mature and consequently lose their capacity to regulate ionic and neurotransmitter homeostasis, changes that strongly promote epileptogenesis [109].

In contrast, TSC microglia display abnormal activation, likely driven by exaggerated agonist-induced Ca^2^⁺ signals. This overactive phenotype has been associated with epileptogenic activity in the TSC brain [41], highlighting their central role in disease pathophysiology. Mechanistically, such pro-epileptogenic effects may arise from heightened phagocytic activity, which is linked to enhanced Ca^2^⁺ signalling, a well-established contributor to epileptogenesis [24, 54, 96, 100, 103]. Since phagocytosis is Ca^2^⁺-dependent and energetically demanding [65, 70], the combination of heightened Ca^2^⁺ dynamics and increased mitochondrial metabolism likely accounts for the elevated phagocytic capacity we observed in TSC iMGLs. Furthermore, mTOR hyperactivation in TSC amplifies this phenotype by regulating mitochondrial biogenesis, lysosomal function, calcium homeostasis, and cytoskeletal remodelling, all of which are essential for microglial activation and phagocytosis [3, 42, 73, 87, 88].

Taken together, we hypothesize that, downstream of TSC mutations and mTOR overactivation, the divergent directions of the remodeling of Ca^2+^ signaling in astrocytes (suppressed) or microglia (enhanced), synergistically contribute to the pathogenetic microenvironment in the TSC brain. This environment is characterized by a reduced homeostatic support, enhanced neuroinflammation and increased phagocytosis, collectively contributing to the process of epileptogenesis.

Interestingly, transcriptomic analysis of TSC brain tissue revealed upregulation of several stage 2 DAM-associated genes (*TREM2, CLEC7A, LPL, AXL, ITGAX,* and *CSF1*). Supporting this, we observed a hyperresponsive, hypermetabolic phenotype in our TSC iMGL cells, which resembles aspects of DAMs, a state previously described in neurodegenerative diseases and epilepsy, and more recently linked to mTOR hyperactivation [17, 43, 86, 88, 108]. Recent studies have demonstrated that DAMs do not represent a single uniform activation state but rather a spectrum of microglial subsets with distinct yet partially overlapping transcriptional programs [59]. As mTORopathies were not included in these analyses, it remains possible that chronic mTOR hyperactivation, as observed in TSC, may bias microglia toward a distinct position within the DAM spectrum. In this context, the hypermetabolic, hyperresponsive, DAM-like phenotype observed in TSC iMGLs may represent a shared microglial activation state that is quantitatively or functionally amplified by disease-specific cues. Whether such DAM-like subsets exert protective or pathogenic effects in TSC, as proposed for other CNS disorders, likely depends on disease stage and microenvironment [17, 21, 40, 43, 88], and warrants further investigation.

In conclusion, our findings demonstrate that even heterozygous pathogenic variants in TSC genes are sufficient to reprogram the metabolic state of microglia, resulting in dysregulated Ca^2^⁺ signalling, increased mitochondrial respiration, altered inflammatory response and enhanced phagocytic activity. This calcium dysregulation not only perturbs intracellular homeostasis but also promotes a shift toward a hypermetabolic, hyperresponsive phenotype. These results highlight the utility of patient-derived iPSC-based microglial models in capturing disease-specific cellular alterations and provide a basis for future mechanistic and therapeutic investigations targeting microglia in TSC. Importantly, because our iMGL system primarily reflects cell-intrinsic processes, future multi-cellular studies using human-derived and/or in vivo models would be important to assess how multicellular network activity shapes microglial behavior and to fully understand the in vivo relevance of these dysregulated phenotypes.

## Supplementary Information

Below is the link to the electronic supplementary material.

Supplementary file1 (DOCX 2691 KB)

## Data Availability

The data that support the findings of this study are available from the corresponding author upon reasonable request.
