# Supplementary material for: TSC-associated microglial hyperactivity: enhanced calcium signaling, metabolism, and phagocytosis
Source: Acta Neuropathol. 2026 Feb 13;151(1):16. doi: 10.1007/s00401-026-02986-8 (PMC12904941; doi:10.1007/s00401-026-02986-8)
Supplement: Supplementary file 1 — Supplementary file1 (DOCX 2691 KB) [file 401_2026_2986_MOESM1_ESM.docx]

**Supplementary data**

**Supplementary Tab. 1** Control and TSC samples used for sc-RNA seq on primary brain tissue. TSC = Tuberous Sclerosis complex (TSC); m = male; f = female; F = Frontal; T = temporal; P = Parietal

|  | **Diagnose** | **Gender** | **Seizure Onset (years)** | **Age (during operation)** | **Duration (years)** | **Area of resection** | **Mutation** |
| --- | --- | --- | --- | --- | --- | --- | --- |
| T8 | TSC | m | 0 | 2 | 2 | F | TSC2 |
| T9 | TSC | m | 0 | 5 | 5 | F | TSC2 |
| T10 | TSC | f | 0 | 8 | 8 | F | TSC2 |
| T11 | TSC | f | 0 | 0 | 0 | T | TSC2 |
| T12 | TSC | f | 0 | 17 | 17 | P | TSC2 |
| C1 | Control | m | NA | 12 | NA | C | NA |
| C2 | Control | f | NA | 16 | NA | C | NA |
| C3 | Control | f | NA | 5 | NA | C | NA |

**Supplementary Tab. 2** iMGL-cell lines used in functional experiments. Donor ID refers to Tab 1.

| **Experiment** | **Cell line(s) used** | **Figure** |
| --- | --- | --- |
| Cytosolic Ca^2+^ response | Control iMGL cells (C1-C4), TSC^+/-^ iMGL cells (T4-T7) | Fig. 4a-c |
| SOCE | Control iMGL cells (C1-C4), TSC^+/-^ iMGL cells (T4-T7) | Fig. 4d-f |
| Mitochondrial Ca^2+^ response | Control iMGL cells (C1-C4), TSC^+/-^ iMGL cells (T4-T7) | Fig. 4g-i |
| qPCR (Ca^2+^ signalling) | Control iMGL cells (C1-C4), TSC^+/-^ iMGL cells (T5-T7) | Fig. 5a |
| Western blot | Control iMGL cells (C1-C4), TSC^+/-^ iMGL cells (T4-T7) | Fig. 5b |
| Oroboros | Control iMGL cells (C1-C4), TSC^+/-^ iMGL cells (T4-T7) | Fig. 6 |
| Phagocytosis assay | Control iMGL cells (C1-C4), TSC^+/-^ iMGL cells (T5-T7) | Fig. 7 |
| qPCR (LPS stimulation) | Control iMGL cells (C1-C4), TSC^+/-^ iMGL cells (T4-T7) | Supplementary Fig. 6 |

**Supplementary Tab. 3** Primers

| Gene | Primer | Sequence |
| --- | --- | --- |
| NFIA | h_NF1A_F  h_NF1A_R | GTGGGGTTCCTCAATCCCAAT  TTGACGTGGTTGGAGGCTTT |
| NANOG | h_NANOG_F  h_NANOG_R | AATGGTGTGACGCAGGGATG  TGCACCAGGTCTGAGTGTTC |
| OCT4 | h_OCT4_F  h_OCT4_R | GAGCAAAACCCGGAGGAGT  TTCTCT TTCGGGCCTGCAC |
| IBA1 | h_IBA1_F  h_IBA1_R | CCAAACCAGGGATTTACAGG  CGTCTAGGAATTGCTTGTTGATCT |
| TMEM119 | h_TMEM119_F  h_TMEM119_R | GGGGAAGGGTTTCTGACG  GCCAGACAATGTGTGAGCAA |
| CX3CR1 | CX3CR1_F  CX3CR1_R | CTCGTCTCTGGTAAAGTCTGAGC  GGGAACTGATCCATGGTGAA |
| IP3R1 | ITPR1_F  ITPR1_R | GAGTTTCAGCCCTCAGTGGAC  GTTCCTTGGGACAAGGCTGT |
| IP3R2 | ITPR2_F  ITPR2_R | TCAGCACCTTGGGGTTAGTG  GTGGTTCCCTTGTTTGGCT |
| IP3R3 | ITPR3_F  ITPR3_R | GTCCTCCGCACTGAGCTTG  GTCATCCACCAGCCCCAAAG |
| SERCA2 | ATP2A2_F  ATP2A2_R | CACCTTCTTCAAACCAAGCCA  CACCTTCTTCAAACCAAGCCA |
| SERCA3 | ATP2A3_F  ATP2A3_R | GGAACCACATGCACGAAGAAA  GATGGCCATTCTGACCTCGG |
| MCU | MCU_F  MCU_R | TCGCTTCCTGGCAGAATTTG  GGGATGGTAGCCTCACAGA |
| MCUB | MCUB_F  MCUB_R | AACTGCAGCCATCTTCACAG  TCTCTCTTTGGACACTGCACA |
| STIM1 | Stim1_F  Stim1_R | GCCTCAGCCATAGTCACAGT  ATGTTACGGACTGCCTCGAA |
| STIM2 | Stim2_F  Stim2_R | GACGGATGCGAGCTTGTG  AAGCATGGTGGACTCAGTGA |
| ORAI1 | Orai 1_F  Orai 1_R | GACCTCGGCTCTGCTCTC  TGATCATGAGCGCAAACAGG |
| ORAI2 | Orai2_F  Orai2_R | CCCTCCTCTCCGGCTTTG  TGATGAGGAGGGCGAACAG |
| IL-1β | IL1β_F  IL1β_R | GCATCCAGCTACGAATCTCC  GAACCAGCATCTTCCTCAGC |
| TNFα | hTNF-alfa_f  hTNF-alfa_r | CCCCAGGGACCTCTCTCTAA  CAGCTTGAGGGTTTGCTACA |
| IL10 | hIL-10_F  hIL-10_R | GATGCCTTCAGCAGAGTGAA  GCAACCCAGGTAACCCTTAAA |

**Supplementary Tab. 4** Antibodies IF staining

| **Primary antibodies** | **Company** | **Cat#** | **Species** | **Dilution (x)** |
| --- | --- | --- | --- | --- |
| TMEM119 | Sigma | HOA051870 | Rabbit | 1000 |
| IBA1 | Invitrogen | MA5-27726 | Mouse | 100 |
| **Secondary antibodies** | **Company** | **Cat#** | **Species** | **Dilution (x)** |
| Phalloidin-iFluor 750 Reagent | Abcam | ab176762 | N.A. | 1:1000 |
| Goat anti-Rabbit IgG (H+L) Cross-Adsorbed Secondary Antibody Alexa 488 | Invitrogen | A-11008 | Goat | 1:2000 |
| Goat anti-Rabbit IgG (H+L) Cross-Adsorbed Secondary Antibody Alexa 568 | Invitrogen | A-11004 | Goat | 1:2000 |

**Supplementary Tab. 5** – Top 15 upregulated and downregulated genes in TSC microglia from primary tissue

| **Upregulated** | | | |
| --- | --- | --- | --- |
|  | **Gene name** | **Log2FoldChange** | **Adjusted p-value** |
| 1 | IGHG1 | 10.0504233 | 0.02989461 |
| 2 | CHIT1 | 9.14058551 | 0.00350923 |
| 3 | CCL4L2 | 8.13026183 | 1.60E-08 |
| 4 | TNFSF18 | 7.73327274 | 4.84E-06 |
| 5 | IGKC | 6.41389244 | 0.02113137 |
| 6 | CCL3L1 | 6.21512094 | 0.00030306 |
| 7 | EGR2 | 6.12790509 | 6.07E-13 |
| 8 | CCL4 | 5.92132232 | 9.30E-16 |
| 9 | IGHM | 5.51122845 | 0.00836856 |
| 10 | TNF | 5.41690176 | 2.01E-09 |
| 11 | SERPINA3 | 5.38043862 | 3.06E-06 |
| 12 | FAM186B | 5.18481513 | 0.00343909 |
| 13 | CXCL8 | 5.08468776 | 0.00188253 |
| 14 | CP | 5.08140779 | 0.00801273 |
| 15 | CXCL3 | 5.04700943 | 0.04023108 |
| **Downregulated** | | | |
|  | **Gene name** | **Log2FoldChange** | **Adjusted p-value** |
| 1 | PNLDC1 | -6.4103298 | 0.00022359 |
| 2 | HSPB1 | -6.1691518 | 8.30E-05 |
| 3 | CSF3 | -6.0394698 | 0.02755639 |
| 4 | BAG3 | -5.7612613 | 0.0004846 |
| 5 | C19orf84 | -5.6375755 | 0.0003 |
| 6 | BPI | -5.6275206 | 0.00805371 |
| 7 | DNAJB1 | -5.5088209 | 0.0003184 |
| 8 | HSPH1 | -5.1158697 | 0.00021493 |
| 9 | NPAS4 | -5.0714835 | 0.02009331 |
| 10 | GPR4 | -4.9330029 | 0.00273378 |
| 11 | SERPINC1 | -4.9130523 | 0.01396204 |
| 12 | SERPINH1 | -4.8992383 | 0.00068505 |
| 13 | DNAJA4 | -4.7458657 | 0.00270515 |
| 14 | HSPA6 | -4.7301278 | 0.00106319 |
| 15 | HSPA1A | -4.7055251 | 0.00046959 |

**
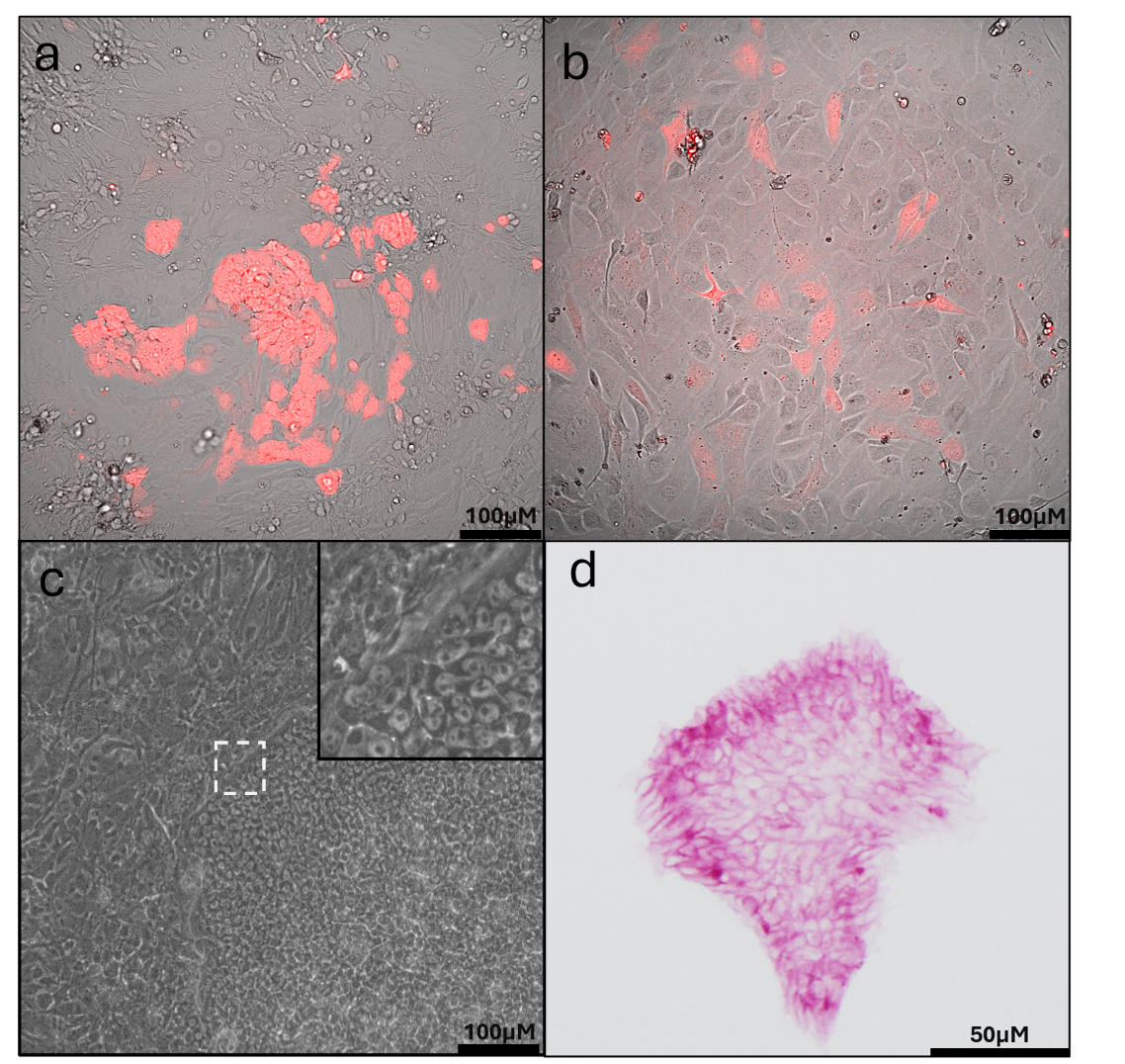
**

**Supplementary Fig. 1** Generation of iPSC cell lines from primary control and TSC astrocyte **a.** Formation of plasmid (dTomato) postive cells and starting formation of iPSC cells in a control cell line 7 days post-transfection. **b.** Plasmid positive cells in an TSC line 7 days post transfection. **c.** Outgrowth of an iPSC colony from an control line 14 days post transfection, with distinct morphology and growing pattern compared to bordering cells. **d.** Alkaline phosphatase (AP) staining of an iPSC colony at passage 10.


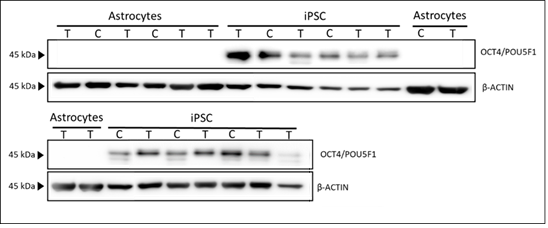


**Supplementary Fig. 2** Western blot of OCT4/POU5F1 in parental (astrocyte) lines (n=10) and generated IPSC lines (n=13), C = control, T = TSC.

**
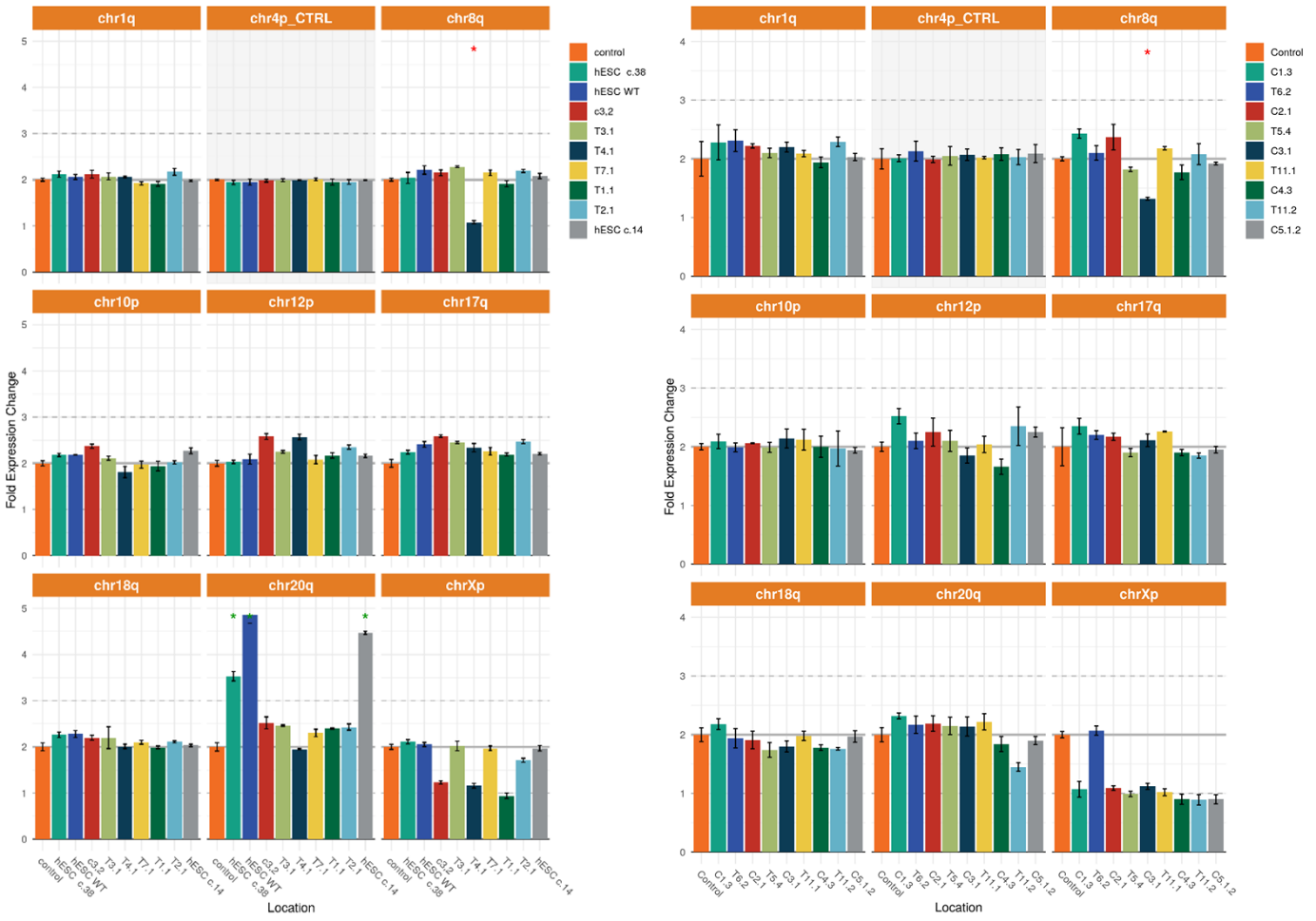
**

**Supplementary Fig. 3** Chromosomal loci copy numbers of frequently mutated sites in iPSC clones at passage 10. Lines and their respective subclone genomic DNA (gDNA) were analyzed by qPCR hybridization probes and referenced to an (internal) control of non-mutated genomic gDNA


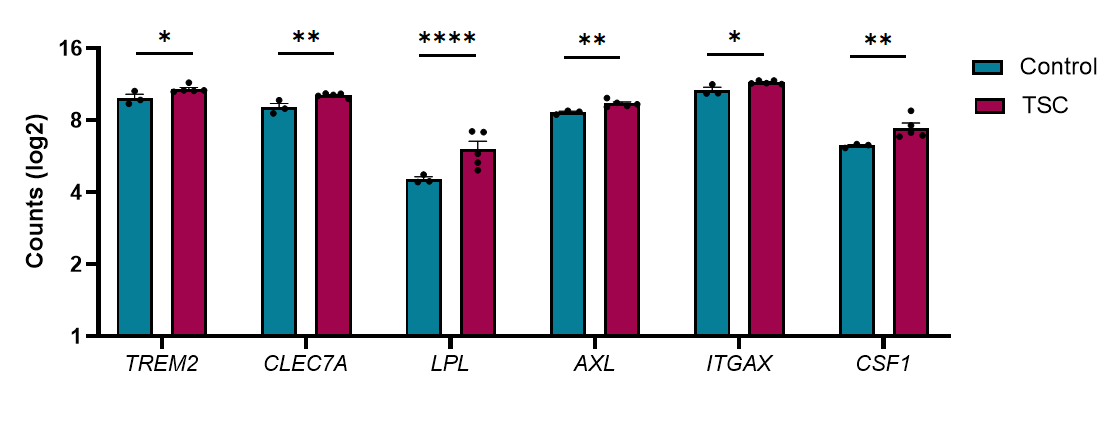


**Supplementary Fig. 4** Differential expression of stage 2 DAM related genes reveal an alteration in TSC microglia using single-cell RNA sequencing on primary tissue (*n*=3 control and *n*=5 TSC**).** In TSC microglia we identified the upregulation of *TREM2* (Log_2_FC = 1,262; p.adj = 0,049); *CLEC7A* (Log_2_FC = 1,557; p.adj = 0,003); *LPL* (Log_2_FC = 4,183; p.adj = 0,0002); *AXL* (Log_2_FC = 1,234; p.adj = 0,002); *ITGAX* (Log_2_FC = 1,166; p.adj = 0,016); *CSF1* (Log_2_FC = 2,434; p.adj = 0,003). Statistical analysis was performed using DESeq2 with Benjamini-Hochberg correction for multiple testing. Data are expressed as mean ± SEM. p-Values indicated by * for p < 0.05, ** for p < 0.01, **** p < 0.0001.

**
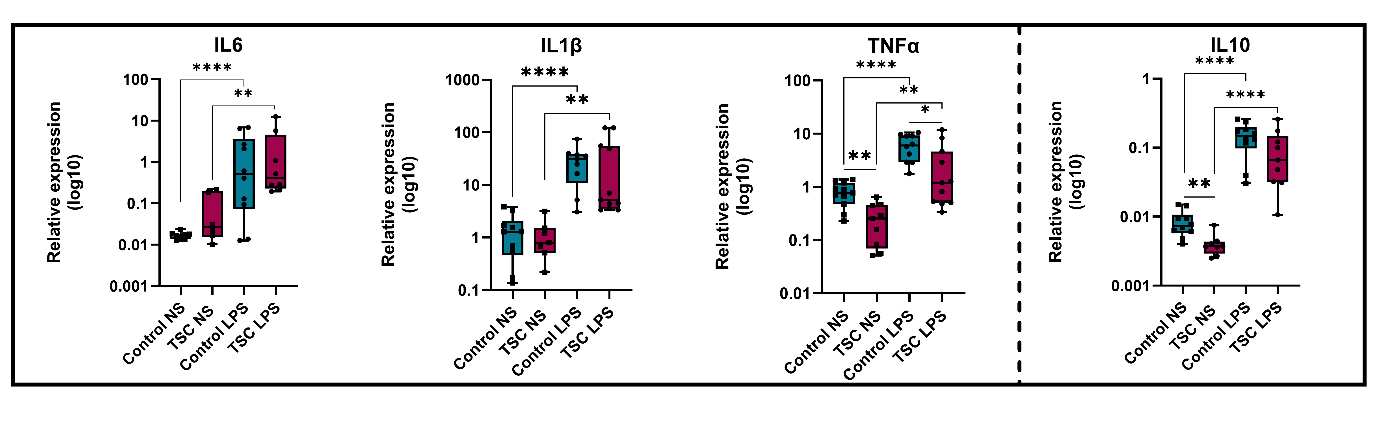
**

**Supplementary Fig. 5 Inflammatory response of control and TSC iMGL-cells.** In response to LPS stimulation, both control and TSC iMGL-cells showed increased expression of the pro-inflammatory markers IL-6, IL-1β, and TNFα, as well as the anti-inflammatory cytokine IL-10. Under non-stimulated conditions, TSC iMGL-cells exhibited lower levels of TNFα and IL-10 compared to control cells. Furthermore, after LPS stimulation, TNFα levels in TSC iMGL-cells remained lower than in controls, while no differences were observed for IL-6 or IL-1β. Data are presented as mean ± SEM for bar graphs (n = 4 control and n = 3 TSC). p-Values indicated by *p < 0.05, **p < 0.01, and ****p < 0.0001. *Abbreviations:* NS, not-stimulated; LPS, lipopolysaccharide.
